# Consumption of macrolides, lincosamides and streptogramins in the community, European Union/European Economic Area, 1997–2017

**DOI:** 10.1093/jac/dkab175

**Published:** 2021-08-01

**Authors:** Niels Adriaenssens, Robin Bruyndonckx, Ann Versporten, Niel Hens, Dominique L Monnet, Geert Molenberghs, Herman Goossens, Klaus Weist, Samuel Coenen, the ESAC-Net study group, Reinhild Strauss, Eline Vandael, Stefana Sabtcheva, Arjana Tambić Andrašević, Isavella Kyriakidou, Jiří Vlček, Ute Wolff Sönksen, Elviira Linask, Emmi Sarvikivi, Karima Hider-Mlynarz, Muna Abu Sin, Flora Kontopidou, Ria Benkő, Gudrun Aspelund, Ajay Oza, Filomena Fortinguerra, Ieva Rutkovska, Rolanda Valintėlienė, Marcel Bruch, Peter Zarb, Stephanie Natsch, Hege Salvesen Blix, Anna Olczak-Pieńkowska, Ana Silva, Gabriel Adrian Popescu, Tomáš Tesař, Milan Čižman, Mayte Alonso Herreras, Vendela Bergfeldt, Berit Müller-Pebody

**Affiliations:** 1 Laboratory of Medical Microbiology, Vaccine & Infectious Disease Institute (VAXINFECTIO), University of Antwerp, Antwerp, Belgium; 2 Centre for General Practice, Department of Family Medicine & Population Health (FAMPOP), University of Antwerp, Antwerp, Belgium; 3 Interuniversity Institute for Biostatistics and statistical Bioinformatics (I-BIOSTAT), Data Science Institute, Hasselt University, Hasselt, Belgium; 4 Centre for Health Economic Research and Modelling Infectious Diseases, Vaccine & Infectious Disease Institute (VAXINFECTIO), University of Antwerp, Antwerp, Belgium; 5 Disease Programmes Unit, European Centre for Disease Prevention and Control, Stockholm, Sweden; 6 Interuniversity Institute for Biostatistics and statistical Bioinformatics (I-BIOSTAT), Catholic University of Leuven, Leuven, Belgium

## Abstract

**Objectives:**

Data on the consumption of macrolides, lincosamides and streptogramins (MLS) in the community were collected from 30 EU/European Economic Area (EEA) countries over two decades. This article reviews temporal trends, seasonal variation, presence of change-points and changes in composition of the main subgroups of MLS.

**Methods:**

For the period 1997–2017, data on consumption of MLS, i.e. ATC group J01F, in the community and aggregated at the level of the active substance, were collected using the WHO ATC/DDD methodology (ATC/DDD index 2019). Consumption was expressed in DDD per 1000 inhabitants per day and in packages per 1000 inhabitants per day. Consumption of MLS was analysed and presented as trends, seasonal variation, presence of change-points and compositional changes, using a classification based on mean plasma elimination half-life for macrolides.

**Results:**

In 2017, consumption of MLS in the community expressed in DDD per 1000 inhabitants per day varied by a factor of 13 between countries with the highest (Greece) and the lowest (Sweden) consumption. Consumption of MLS did not change significantly up to 2003, after which it significantly increased up to 2007. No significant change was observed after 2007. Consumption of MLS showed high seasonal variation. The proportional consumption of long-acting macrolides significantly increased over time compared with that of intermediate-acting macrolides, and proportional consumption of the latter increased compared with that of short-acting macrolides.

**Conclusions:**

Consumption of MLS did not change significantly over time during 2007–2017, while the proportional consumption of long-acting macrolides increased. Seasonal variation remained high, which suggests that MLS are still prescribed inappropriately in many countries.

## Introduction

This article presents data from the European Surveillance of Antimicrobial Consumption Network (ESAC-Net,^1^ formerly ESAC) on community (i.e. primary care sector) consumption of macrolides, lincosamides and streptogramins (MLS) for 30 EU/European Economic Area (EEA) countries in 2017 (Table 1). It updates previous ESAC studies published in 2006 and 2011, and in doing so it provides updated comparable and reliable information on antibiotic consumption that can aid in fighting the global problem of antimicrobial resistance.^2^^,^^3^ In 2017, consumption of MLS represented 16.1% of antibiotic consumption in the community.^4^ As in the previous series, a classification of macrolides based on their mean plasma elimination half-life was adopted. The objective of this study was to analyse temporal trends, seasonal variation and the presence of change-points in consumption of MLS in the community for the period 1997–2017, as well as to analyse the composition of consumption of MLS over time.

**Table 1.** Classification of macrolides, lincosamides and streptogramins (J01F; ATC/DDD index 2019)

Macrolides					
Short-acting		Intermediate-acting		Long-acting	
J01FA01	Erythromycin^a^	J01FA06	Roxithromycin	J01FA10	**Azithromycin** ^a^
J01FA02	Spiramycin	J01FA07	Josamycin	J01FA13	*Dirithromycin*
J01FA03	Midecamycin	J01FA09	**Clarithromycin** ^a^		
J01FA05	*Oleandomycin* ^b^	J01FA14	*Flurithromycin*		
J01FA08	*Troleandomycin* ^b^	J01FA15	Telithromycin		
J01FA11	Miocamycin	J01FA16	*Solithromycin* ^c^		
J01FA12	*Rokitamycin*				
Lincosamides					

J01FF01	**Clindamycin** ^a^				
J01FF02	Lincomycin				
Streptogramins					

J01FG01	Pristinamycin				
J01FG02	*Quinupristin/dalfopristin* ^b^				

**Bold type** indicates that consumption was part of the top 90% of the community consumption of macrolide, lincosamide and streptogramin (MLS) antibiotics (J01F) in 28 EU/EEA countries in 2017; *Italic type* indicates that no consumption of this MLS antibiotic was reported in 28 EU/EEA countries in 2017.

a Consumption was part of the top 90% of the community consumption of MLS antibiotics (J01F) in 30 EU/EEA countries in 2009.

b No consumption of this MLS antibiotic was reported in 30 EU/EEA countries in 2009.

c This MLS antibiotic was not included in the ATC/DDD index in 2009 and was therefore not reported in 2009.

## Methods

The methods for collecting and analysing the data are described in the introductory article of this series.^5^ In summary, data on consumption of MLS, i.e. ATC group J01F and aggregated at the level of the active substance, were collected using the WHO ATC/DDD methodology (ATC/DDD index 2019^5^) and expressed in DDD per 1000 inhabitants per day. In addition, where data were available, consumption of MLS was also expressed in packages per 1000 inhabitants per day. For macrolides, a classification according to the mean plasma elimination half-life, subdividing macrolides into short- (half-life <4 h), intermediate- (half-life from 4 to 24 h) and long-acting macrolides (half-life >24 h), was used to assess macrolide consumption in the community in more detail (Table 1).

The evolution of the number of DDD per package over time was assessed using a linear mixed model. The temporal trend, seasonal variation and presence of change-points in consumption of MLS were assessed using a non-linear change-point mixed model fitted to quarterly data expressed in DDD per 1000 inhabitants per day from 1997 to 2017.^6^ The relative proportions of the main subgroups were assessed through a compositional data analysis modelling yearly data expressed in DDD per 1000 inhabitants per day from 1997 to 2017.^7^

## Results

An overview of consumption of MLS (ATC J01F) in the community, expressed in DDD and packages per 1000 inhabitants per day for all participating countries between 1997 and 2017 is available as Supplementary data at *JAC* Online (Tables S1 and S2, respectively).

### Consumption of MLS in the community in 2017

In 2017, three substances accounted for 90% of the consumption of MLS in the community expressed in DDD per 1000 inhabitants per day: clarithromycin (45.8% in 2017 compared with 54.2% in 2009), azithromycin (35.5% in 2017 compared with 23.3% in 2009) and clindamycin (10.3% in 2017 compared with 6.6% in 2009) (Table 1).

**Figure 1.**
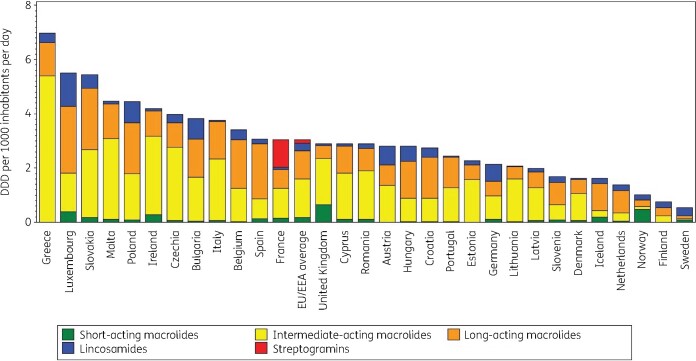
Consumption of macrolides, lincosamides and streptogramins (ATC J01F) in the community, expressed in DDD (ATC/DDD index 2019) per 1000 inhabitants per day, 30 EU/EEA countries, 2017. For Czechia, 2015 data are used. For Slovakia, 2016 data are used. For Cyprus and Romania, total care data, i.e. community and hospital sector combined, are used.

Figure 1 shows the consumption of MLS in the community expressed in DDD per 1000 inhabitants per day for 30 EU/EEA countries in 2017. Consumption of MLS in the community varied by a factor of 13 between countries with the highest (6.98 DDD per 1000 inhabitants per day in Greece) and the lowest (0.54 DDD per 1000 inhabitants per day in Sweden) consumption, which was lower than in 2009 (factor of 18, from 11.54 DDD per 1000 inhabitants per day in Greece to 0.63 DDD per 1000 inhabitants per day in Sweden). Inter-country variations for macrolides (J01FA) were high for short-acting macrolides, intermediate-acting macrolides and long-acting macrolides. While lincosamide (J01FF; mainly clindamycin) consumption was reported in all countries, streptogramin (J01FG, pristinamycin) consumption in the community was only reported in France (Table S1).

In 2017, short-acting macrolides (mainly erythromycin) were the most consumed subgroup of MLS in Norway (>40% of consumption of MLS in the community), and accounted for >20% of consumption in the United Kingdom and >10% in Iceland and Sweden. Intermediate-acting macrolides (mainly clarithromycin, except Denmark mainly roxithromycin) were the most consumed subgroup of MLS in Austria, Bulgaria, Cyprus (total care data, i.e. community and hospital sector combined), Czechia, Denmark, Estonia, France, Germany, Greece, Ireland, Italy, Latvia, Lithuania, Malta, Portugal, Romania (total care data), Slovakia and the United Kingdom. Clarithromycin and roxithromycin consumption represented >30% of consumption of MLS in Belgium, Croatia, Hungary, Poland and Slovenia. Long-acting macrolides were the most consumed subgroup of MLS in Belgium, Croatia, Finland, Hungary, Iceland, Luxembourg, the Netherlands, Poland, Slovenia and Spain. Compared with 2009, the proportional consumption of long-acting macrolides increased in most countries. Lincosamides were the most consumed MLS in Sweden and represented >20% of the consumption of MLS in the community in Austria, Finland, Germany, Hungary and Luxembourg.

**Figure 2.**
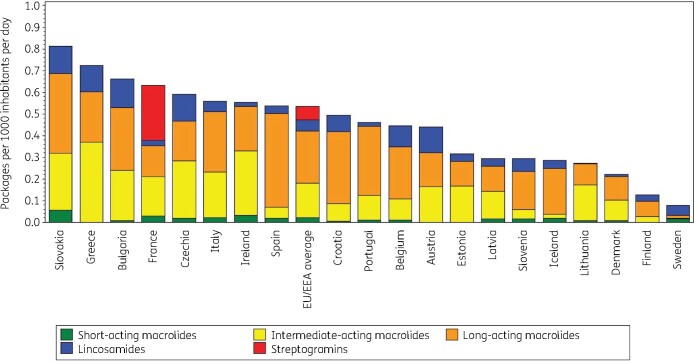
Consumption of macrolides, lincosamides and streptogramins (ATC J01F) in the community, expressed in packages per 1000 inhabitants per day, 20 EU/EEA countries, 2017. For Czechia, 2015 data are used. For Slovakia, 2016 data are used. For Cyprus and Romania, total care data, i.e. community and hospital sector combined, are used.

Figure 2 shows consumption of MLS in the community expressed in packages per 1000 inhabitants per day for 20 EU/EEA countries in 2017. France ranked 9th for its consumption of MLS in DDD per 1000 inhabitants per day and 4th in packages per 1000 inhabitants per day (Table 2). The number of DDD per package ranged from 4.8 in France to 9.6 in Greece. In the EU/EEA countries, the number of DDD per package did not change significantly over time during 1997–2017.

**Table 2.** Ranking of consumption of macrolides, lincosamides and streptogramins (ATC J01F) in the community, expressed in DDDs or packages per 1000 inhabitants per day, 20 EU/EEA countries, 2017

Country	Slovakia	Greece	Bulgaria	France	Czechia	Italy	Ireland	Spain	Croatia	Portugal	Belgium	Austria	Estonia	Latvia	Slovenia	Iceland	Lithuania	Denmark	Finland	Sweden
Ranking for packages per 1000 inhabitants per day	1	2	3	4	5	6	7	8	9	10	11	12	13	14	15	16	17	18	19	20
Ranking for DDD per 1000 inhabitants per day	2	1	5	9	4	6	3	8	11	12	7	10	13	15	16	18	14	17	19	20
Number of DDD per package	6.7	9.6	5.8	4.8	6.7	6.7	7.6	5.7	5.5	5.3	7.7	6.4	7.2	6.7	5.7	5.6	7.6	7.4	5.9	7.0

For Czechia, 2015 data are used. For Slovakia, 2016 data are used. For Cyprus and Romania, total care data, i.e. community and hospital sector combined, are used.

### Longitudinal data analysis, 1997–2017

**Figure 3.**
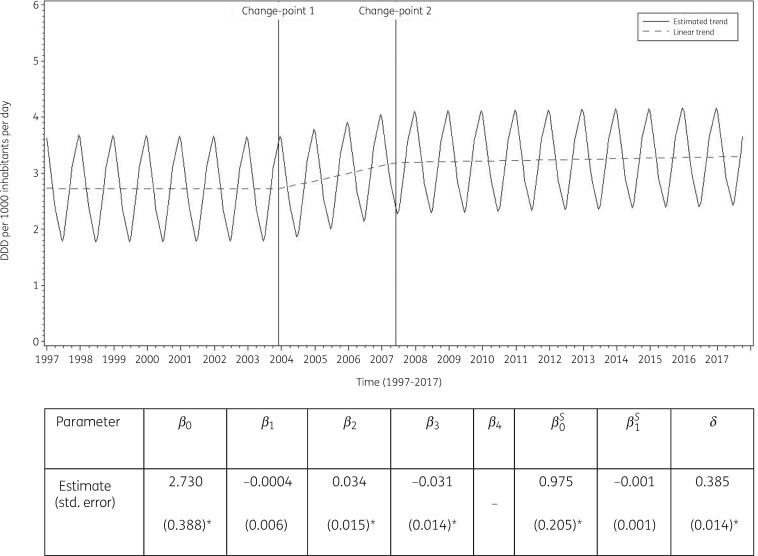
Estimated trend (solid line) and linear trend (dashed line) of consumption of macrolides, lincosamides and streptogramins (ATC J01F) in the community based on quarterly data, 25 EU/EEA countries, 1997–2017. *β_0_*, predicted consumption in the first quarter of 1997; *β_1_*, predicted increase (if positive)/decrease (if negative) in consumption per quarter; *β_2_*, predicted difference in slope after versus before the first change-point; *β_3_*, predicted difference in slope after versus before the second change-point; *β_4_*, predicted difference in slope after versus before the third change-point; *β_0_^S^*, predicted amplitude of the upward winter and downward summer peak in consumption; *β_1_^S^*, predicted increase (if positive)/decrease (if negative) of the amplitude of the upward winter and downward summer peak in consumption per quarter; *δ*, shift in timing of the upward winter and downward summer peak from one year to another. An asterisk indicates that the result was statistically significant at significance level 0.05.

The best model fit was obtained for a model including two change-points: one in the last quarter of 2003 and another in the second quarter of 2007. The final model fits the observed data well (Figure S1). The longitudinal data analysis estimated an average consumption of MLS in the EU/EEA of 2.73 (SE 0.39) DDD per 1000 inhabitants per day in the first quarter of 1997. Consumption of MLS did not change significantly (−0.0004, SE 0.006, DDD per 1000 inhabitants per day per quarter) between 1997 and the last quarter of 2003. After this first change-point, consumption of MLS significantly increased (+0.033, SE 0.015, DDD per 1000 inhabitants per day per quarter). After the second change point, consumption of MLS did not change significantly (+0.0028, SE 0.017, DDD per 1000 inhabitants per day per quarter; Figure 3). The longitudinal analysis showed significant seasonal variation with an amplitude of 0.98 (SE 0.21) DDD per 1000 inhabitants per day, which did not change significantly over time (−0.0006, SE 0.0006, DDD per 1000 inhabitants per day per quarter).

Based on the fitted model, consumption of MLS in 2017 was significantly higher than average in Austria, Belgium, Greece and Italy, and significantly lower than average in Croatia, Estonia, Finland, Iceland, Lithuania, the Netherlands, Poland and Sweden (observed profiles shown in Figures S2 and S3). The seasonal variation was significantly larger than average in Greece, Italy and Slovakia and significantly smaller than average in Denmark, Finland, Iceland, the Netherlands, Sweden and the United Kingdom. The decrease in consumption of MLS between 1997 and the last quarter of 2003 was significantly larger than average in Austria, Belgium, Spain and the United Kingdom. The increase in consumption of MLS between 2004 and the second quarter of 2007 was significantly larger than average in Croatia, Estonia, Ireland, Slovakia and Lithuania.

Table S1 provides an overview of the trends in consumption of MLS in the participating countries between 1997 and 2017. Consumption of MLS decreased in most countries, in particular in Greece. Conversely, an increase in consumption of MLS was observed for Luxembourg, Spain and Latvia. The seasonal variation in consumption of MLS is shown in Figures S2 and S3. The mean consumption of MLS in the first and fourth quarters (winter) was >50% higher than the mean consumption of MLS in the second and third quarters (summer) in Estonia, Italy and Latvia; >40% in Belgium, Croatia, and Portugal; and >30% in Germany and Luxembourg. Clarithromycin consumption was >50% higher in winter quarters than in summer quarters in Belgium, Croatia, Estonia, Finland, Hungary, Italy, Latvia and Luxembourg. Azithromycin consumption was >50% higher in winter quarters than in summer quarters in Croatia, Estonia, Italy, Latvia, Luxembourg and Portugal.

### Compositional data analysis, 1997–2009

The proportional consumption of long-acting macrolides and streptogramins significantly increased over time relative to that of short- and intermediate-acting macrolides and lincosamides. The proportional consumption of streptogramins did not change significantly relative to that of long-acting macrolides. The proportional consumption of intermediate-acting macrolides and lincosamides significantly increased relative to that of short-acting macrolides (Table 3).

**Table 3.** Change in composition of the consumption of macrolides, lincosamides and streptogramins (ATC J01F) in the community, expressed in DDD (ATC/DDD index 2019) per 1000 inhabitants per day, 30 EU/EEA countries, as a function of time during 1997–2017

	SAM	IAM	LAM	J01FF	J01FG
SAM		**−0.1276**	**−0.1895**	**−0.1824**	**−0.2271**
IAM	**0.1276**		**−0.0619**	**−0.0549**	**−0.0995**
LAM	**0.1895**	**0.0619**		0.0071	−0.0376
J01FF	**0.1824**	**0.0549**	−0.0071		**−0.0447**
J01FG	**0.2271**	**0.0995**	0.0376	**0.0447**	

Values are estimated changes in the log ratio of the row versus column subgroup of antibiotics with increasing time. Bold type indicates a statistically significant effect; positive values represent an increase and negative values represent a decrease.

SAM, short-acting macrolides; IAM, intermediate-acting macrolides; LAM, long-acting macrolides; J01FF, lincosamides; J01FG, streptogramins.

Trends of proportional consumption in individual countries are shown in Figure S4. When comparing the composition of macrolide consumption in 2017 with that in 2009, the proportion of short-acting macrolides decreased in all participating countries, mainly as the result of decreasing erythromycin consumption. The largest decreases were observed for the United Kingdom (−35.47%), Sweden (−28.01%), Romania (−26.08%; total care data; coverage in 2009 limited to 30%–40%) and Denmark (−23.15%). The proportion of intermediate-acting macrolides decreased in most of the participating countries. Decreases >10% were observed for 13 out of 30 participating countries, with the largest decreases observed for Luxembourg (−28.84%), the Netherlands (−26.12%), Belgium (−24.07%), Hungary (−22.74%) and France (−20.40%). However, increases were also observed, with the largest increases being reported for the United Kingdom (+23.37%), Denmark (+9.80%) and Lithuania (+4.17%). The proportion of long-acting macrolides increased in all countries except Slovenia (−3.12%). Increases >10% were observed for 17 out of 30 participating countries, with the largest increases observed for Latvia (+29.09%), Belgium (+25.64%), Iceland (+24.51%), the Netherlands (+24.46%), Hungary (+24.08%) and Luxembourg (+21.06%).

The proportion of lincosamides increased for most countries, with increases >10% being reported for Germany (+19.04%), Sweden (+15.71%), Finland (+13.20%) and Luxembourg (+11.90%). Streptogramins were only consumed in France, where their proportion increased (+8.00%).

## Discussion

This study describes consumption of MLS in the community in the EU/EEA and found that the observed increasing trend of consumption of MLS up to 2009 in many countries continued afterwards. However, in more than half of the countries we observed a lower consumption of MLS in DDD per 1000 inhabitants per day in 2017 than in 2009. Greece was still, in 2017, the EU/EEA country with the highest consumption of MLS. We did not observe any important shifts in the ranking of EU/EEA countries when using packages per 1000 inhabitants per day instead of DDD per 1000 inhabitants per day as consumption metric.

In 2017, the consumption of MLS ranged from 4.77% in Sweden to 26.29% in Luxembourg.^4^ In European countries that are not part of the ESAC-Net but covered by the WHO Europe Antimicrobial Medicines Consumption (AMC) Network, the inter-country variation was less pronounced, i.e. from 4.8% (Kyrgyzstan) to 15.9% (Russian Federation) of the total (i.e. community and hospital sector combined) consumption.^8^

Three substances, i.e. clarithromycin, azithromycin and clindamycin, represented more than 90% of the community consumption of MLS in the EU/EEA. For most EU/EEA countries, the proportional consumption of long-acting macrolides (i.e. azithromycin) increased at the expense of that of the other subgroups. Only in Norway did the consumption of short-acting macrolides (i.e. erythromycin) remain predominant. In comparison with 2009,^3^ spiramycin only represented <1% of the community consumption of MLS in 2017. Given that total macrolide consumption did not change significantly after 2007, this implies that consumption of one subgroup was merely replaced by consumption of another subgroup, rather than consumption being reduced overall.

Both azithromycin and clarithromycin use have been shown to increase the proportion of macrolide-resistant streptococci in healthy volunteers but, while this proportion was higher after azithromycin use than clarithromycin use, only clarithromycin use selected for the *erm*(B) gene that confers high-level macrolide resistance.^9^ The observed increasing proportional consumption of azithromycin in the EU/EEA corresponds to that reported for the USA.^10–12^ The current European guidelines for the management of community-acquired lower respiratory tract infections in adults recommend amoxicillin as first-line therapy with macrolides being reserved for patients with penicillin allergy and regions where macrolide resistance in pneumococcal isolates is low.^13^ Yet, the seasonal variation in the consumption of MLS in the EU/EEA remained substantial compared with that of overall antibiotic consumption (ATC J01) and other main groups of antibiotics.^4^^,^^14–17^ While a minimal amount of seasonal variation could be associated with seasonality in bacterial pathogens, the extent of the observed seasonality suggests inappropriate prescribing during the winter in many countries.

In Belgium, maintaining macrolide consumption below a critical threshold was associated with both maintenance of a low prevalence of macrolide-resistant *Streptococcus pyogenes* and emergence of milder resistance mechanisms with lower fitness costs.^18^^,^^19^ In addition, Megraud *et al*.^20^ found a significant association between the use of specifically long-acting macrolides and clarithromycin resistance in *Helicobacter pylori*. A high rate of clarithromycin resistance no longer allows for its empirical use as standard regimen for the treatment of *H. pylori* infections. Current antibiotic guidelines for the treatment of *H. pylori* infections recommend bismuth quadruple therapy (A02BD08) in case of high macrolide resistance rates.

France is the only country using streptogramins (i.e. pristinamycin). Pristinamycin is included in guidelines for the treatment of sinusitis, exacerbation of chronic bronchitis, pneumonia and skin infections, mostly in case of penicillin allergy.^21–23^ Linking antibiotic prescribing data with indications for these prescriptions would be helpful to achieve a better understanding of inter-country variations and trends in the consumption of MLS.

With the exception of clindamycin, all MLS (ATC J01F) are listed as Watch or Reserve group antibiotics in the 2019 WHO Access, Watch or Reserve (AWaRe) classification list.^24^ The continuous monitoring of the consumption of MLS in the community can help to assess the impact of future interventions promoting better use of these antibiotics.

For a more-detailed discussion on the limitations of the collected data, we refer to the article on antibacterials for systemic use, included in this series.^5^ For a discussion on the limitations of the statistical approach used in this study and potential explanations for the common change-points detected through these analyses, we refer to the tutorial included in this series.^6^

In conclusion, the increase in the consumption of MLS in the EU/EEA ended in 2007, after which consumption of MLS remained stable. The proportional consumption of long-acting macrolides, i.e. azithromycin, increased over time during 1997–2017. Seasonal variation remained high, which suggests that MLS are still prescribed inappropriately in the community in many countries.

## Supplementary Material

dkab175_Supplementary_Data

## Supplementary data

Tables S1 and S2 and Figures S1 to S4 are available as Supplementary data at *JAC* Online.
