# Supplementary material for: Consumption of macrolides, lincosamides and streptogramins in the community, European Union/European Economic Area, 1997–2017
Source: J Antimicrob Chemother. 2021 Aug 1;76(Suppl 2):ii30–6. doi: 10.1093/jac/dkab175 (PMC8314107; doi:10.1093/jac/dkab175)
Supplement: dkab175_Supplementary_Data [file dkab175_supplementary_data.docx]

**Supplementary data**

**Table S1. Consumption of macrolides, lincosamides and streptogramins (ATC J01F) in the community, expressed in DDD (ATC/DDD index 2019) per 1000 inhabitants per day, 30 EU/EEA countries, 1997-2017.**

| **Country** | **1997** | **1998** | **1999** | **2000** | **2001** | **2002** | **2003** | **2004** | **2005** | **2006** | **2007** | **2008** | **2009** | **2010** | **2011** | **2012** | **2013** | **2014** | **2015** | **2016** | **2017** |
| --- | --- | --- | --- | --- | --- | --- | --- | --- | --- | --- | --- | --- | --- | --- | --- | --- | --- | --- | --- | --- | --- |
| **Austria** | **-** | **3.50** | **3.73** | **3.22** | **2.99** | **2.91** | **2.89** | **3.04** | **3.43** | **3.41** | **3.59** | **3.66** | **3.93** | **3.57** | **3.39** | **3.19** | **3.59** | **3.04** | **3.06** | **2.74** | **2.81** |
| SAM | **-** | 0.21 | 0.16 | 0.12 | 0.11 | 0.08 | 0.07 | 0.07 | 0.07 | 0.06 | 0.05 | 0.05 | 0.04 | 0.01 | 0.01 | 0.01 | 0.01 | 0.01 | 0.01 | 0.01 | <0.01 |
| IAM | **-** | 2.41 | 2.72 | 2.30 | 2.10 | 2.04 | 2.14 | 2.09 | 2.41 | 2.37 | 2.39 | 2.36 | 2.49 | 2.22 | 2.05 | 1.85 | 2.01 | 1.64 | 1.63 | 1.39 | 1.35 |
| LAM | **-** | 0.60 | 0.55 | 0.48 | 0.44 | 0.42 | 0.46 | 0.45 | 0.49 | 0.47 | 0.57 | 0.63 | 0.73 | 0.63 | 0.62 | 0.62 | 0.76 | 0.69 | 0.74 | 0.68 | 0.75 |
| Lincosamides | **-** | 0.27 | 0.30 | 0.32 | 0.34 | 0.37 | 0.21 | 0.44 | 0.46 | 0.51 | 0.57 | 0.62 | 0.68 | 0.71 | 0.70 | 0.71 | 0.81 | 0.70 | 0.69 | 0.67 | 0.69 |
| Streptogramins | **-** | **-** | **-** | **-** | **-** | **-** | **-** | **-** | **-** | **-** | **-** | **-** | **-** | **-** | **-** | **-** | **-** | **-** | **-** | **-** | **-** |
| **Belgium** | **3.39** | **3.78** | **3.72** | **3.66** | **3.33** | **3.22** | **3.00** | **2.33** | **2.51** | **2.46** | **2.63** | **2.81** | **2.94** | **2.92** | **3.18** | **3.40** | **3.34** | **3.40** | **3.63** | **3.62** | **3.41** |
| SAM | 0.43 | 0.55 | 0.48 | 0.40 | 0.34 | 0.30 | 0.25 | 0.17 | 0.15 | 0.12 | 0.14 | 0.16 | 0.11 | 0.10 | 0.09 | 0.08 | 0.06 | 0.06 | 0.05 | 0.03 | 0.02 |
| IAM | 2.28 | 2.56 | 2.48 | 2.44 | 2.12 | 2.01 | 1.89 | 1.54 | 1.72 | 1.68 | 1.72 | 1.74 | 1.76 | 1.68 | 1.75 | 1.76 | 1.63 | 1.55 | 1.54 | 1.44 | 1.22 |
| LAM | 0.47 | 0.45 | 0.54 | 0.62 | 0.67 | 0.72 | 0.67 | 0.44 | 0.43 | 0.44 | 0.53 | 0.65 | 0.79 | 0.84 | 1.02 | 1.21 | 1.31 | 1.44 | 1.68 | 1.77 | 1.79 |
| Lincosamides | 0.21 | 0.21 | 0.21 | 0.21 | 0.20 | 0.20 | 0.19 | 0.19 | 0.21 | 0.22 | 0.23 | 0.26 | 0.28 | 0.30 | 0.31 | 0.34 | 0.35 | 0.35 | 0.37 | 0.38 | 0.38 |
| Streptogramins | <0.01 | <0.01 | <0.01 | <0.01 | <0.01 | <0.01 | **-** | **-** | **-** | **-** | **-** | **-** | **-** | **-** | **-** | **-** | **-** | **-** | **-** | **-** | **-** |
| **Bulgaria** | **-** | **-** | ***0.35*** | ***0.72*** | ***0.60*** | ***0.74*** | ***1.44*** | ***1.02*** | ***1.36*** | **1.81** | **2.60** | **3.16** | **3.16** | **3.16** | **3.41** | **3.16** | **3.44** | **3.93** | **3.87** | **3.67** | **3.82** |
| SAM | **-** | **-** | *0.19* | *0.41* | *0.31* | *0.33* | *0.72* | *0.31* | *0.40* | 0.42 | 0.34 | 0.27 | 0.20 | 0.16 | 0.15 | 0.11 | 0.09 | 0.08 | 0.06 | 0.05 | 0.04 |
| IAM | **-** | **-** | *0.06* | *0.08* | *0.05* | *0.12* | *0.31* | *0.26* | *0.28* | 0.64 | 1.23 | 1.55 | 1.60 | 1.57 | 1.71 | 1.50 | 1.62 | 1.79 | 1.71 | 1.55 | 1.62 |
| LAM | **-** | **-** | *0.01* | *0.07* | *0.12* | *0.16* | *0.23* | *0.30* | *0.42* | 0.55 | 0.74 | 0.91 | 0.88 | 0.91 | 0.99 | 0.90 | 1.00 | 1.39 | 1.39 | 1.35 | 1.41 |
| Lincosamides | **-** | **-** | *0.08* | *0.17* | *0.12* | *0.13* | *0.17* | *0.15* | *0.26* | 0.20 | 0.30 | 0.42 | 0.47 | 0.51 | 0.57 | 0.65 | 0.72 | 0.68 | 0.71 | 0.72 | 0.74 |
| Streptogramins | **-** | **-** | ***-*** | ***-*** | ***-*** | *<0.01* | ***-*** | ***-*** | ***-*** | **-** | **-** | **-** | **-** | **-** | **-** | **-** | **-** | **-** | **-** | **-** | **-** |
| **Croatia** | **-** | **-** | **-** | **-** | **1.93** | **1.96** | **2.16** | **2.32** | **2.88** | **2.82** | **3.49** | **3.43** | **3.43** | **3.04** | **2.89** | **2.97** | **2.80** | **2.91** | **3.10** | **2.71** | **2.75** |
| SAM | **-** | **-** | **-** | **-** | 0.10 | 0.14 | 0.13 | 0.13 | 0.11 | 0.10 | 0.10 | 0.09 | 0.07 | 0.06 | 0.06 | 0.03 | 0.02 | 0.02 | 0.02 | 0.02 | 0.02 |
| IAM | **-** | **-** | **-** | **-** | 0.17 | 0.16 | 0.27 | 0.57 | 0.86 | 0.97 | 1.46 | 1.42 | 1.26 | 1.27 | 1.11 | 1.08 | 0.91 | 0.89 | 0.96 | 0.85 | 0.87 |
| LAM | **-** | **-** | **-** | **-** | 1.48 | 1.49 | 1.54 | 1.38 | 1.65 | 1.49 | 1.68 | 1.64 | 1.82 | 1.62 | 1.47 | 1.61 | 1.58 | 1.68 | 1.79 | 1.51 | 1.52 |
| Lincosamides | **-** | **-** | **-** | **-** | 0.17 | 0.17 | 0.23 | 0.24 | 0.26 | 0.25 | 0.26 | 0.28 | 0.27 | 0.09 | 0.25 | 0.26 | 0.28 | 0.32 | 0.33 | 0.33 | 0.34 |
| Streptogramins | **-** | **-** | **-** | **-** | **-** | **-** | **-** | **-** | **-** | **-** | **-** | **-** | **-** | **-** | **-** | **-** | **-** | **-** | **-** | **-** | **-** |
| **Cyprus** | **-** | **-** | **-** | **-** | **-** | **-** | **-** | **-** | **-** | ***3.33*** | ***3.59*** | ***3.55*** | ***3.98*** | ***2.90*** | ***3.11*** | ***2.92*** | ***2.67*** | ***2.46*** | ***3.27*** | ***2.79*** | ***2.89*** |
| SAM | **-** | **-** | **-** | **-** | **-** | **-** | **-** | **-** | **-** | *0.36* | *0.30* | *0.31* | *0.21* | *0.16* | *0.20* | *0.18* | *0.10* | *0.10* | *0.10* | *0.13* | *0.10* |
| IAM | **-** | **-** | **-** | **-** | **-** | **-** | **-** | **-** | **-** | *2.37* | *2.56* | *2.52* | *2.85* | *1.99* | *2.27* | *1.86* | *1.67* | *1.51* | *2.10* | *1.56* | *1.71* |
| LAM | **-** | **-** | **-** | **-** | **-** | **-** | **-** | **-** | **-** | *0.55* | *0.68* | *0.66* | *0.87* | *0.71* | *0.59* | *0.82* | *0.83* | *0.78* | *0.98* | *1.00* | *0.99* |
| Lincosamides | **-** | **-** | **-** | **-** | **-** | **-** | **-** | **-** | **-** | *0.05* | *0.05* | *0.05* | *0.06* | *0.05* | *0.06* | *0.06* | *0.07* | *0.07* | *0.09* | *0.10* | *0.09* |
| Streptogramins | **-** | **-** | **-** | **-** | **-** | **-** | **-** | **-** | **-** | ***-*** | ***-*** | ***-*** | ***-*** | ***-*** | ***-*** | ***-*** | ***-*** | ***-*** | ***-*** | ***-*** | ***-*** |
| Country, community consumption of macrolides, lincosamides and streptogramins (J01F); SAM, consumption of short-acting macrolides; IAM, consumption of intermediate-acting macrolides; LAM, consumption of  long-acting macrolides; **-**, no consumption reported; Numbers reported in *italic* are total care data, i.e. community and hospital sector combined; ^a^Data for Romania have a coverage in 2009 limited to 30-40%; ^b^Data for Spain include private prescriptions from 2016 onwards. | | | | | | | | | | | | | | | | | | | | | |
| **Czechia** | **-** | **2.47** | **2.46** | **-** | **-** | **-** | **2.50** | **2.68** | **3.36** | **2.87** | **3.20** | **3.33** | **3.66** | **3.47** | **3.64** | **3.48** | **3.74** | **3.81** | **3.98** | **-** | **-** |
| SAM | **-** | 0.40 | 0.40 | **-** | **-** | **-** | 0.16 | 0.12 | 0.12 | 0.23 | 0.05 | 0.15 | 0.14 | 0.12 | 0.11 | 0.09 | 0.10 | 0.07 | 0.06 | **-** | **-** |
| IAM | **-** | 1.64 | 1.60 | **-** | **-** | **-** | 1.74 | 1.93 | 2.53 | 1.98 | 2.37 | 2.25 | 2.50 | 2.36 | 2.51 | 2.42 | 2.57 | 2.59 | 2.71 | **-** | **-** |
| LAM | **-** | 0.41 | 0.41 | **-** | **-** | **-** | 0.51 | 0.52 | 0.58 | 0.52 | 0.63 | 0.74 | 0.81 | 0.77 | 0.78 | 0.73 | 0.81 | 0.85 | 0.90 | **-** | **-** |
| Lincosamides | **-** | 0.03 | 0.05 | **-** | **-** | **-** | 0.10 | 0.12 | 0.13 | 0.13 | 0.14 | 0.19 | 0.21 | 0.22 | 0.24 | 0.24 | 0.26 | 0.29 | 0.31 | **-** | **-** |
| Streptogramins | **-** | **-** | **-** | **-** | **-** | **-** | **-** | **-** | **-** | **-** | **-** | **-** | **-** | **-** | **-** | **-** | **-** | **-** | **-** | **-** | **-** |
| **Denmark** | **2.04** | **2.28** | **2.18** | **2.04** | **2.10** | **2.16** | **2.14** | **2.24** | **2.34** | **2.32** | **2.45** | **2.34** | **2.26** | **2.38** | **2.66** | **2.25** | **1.84** | **1.84** | **1.84** | **1.80** | **1.62** |
| SAM | 1.18 | 1.25 | 1.11 | 1.11 | 1.09 | 1.10 | 1.01 | 0.95 | 0.83 | 0.82 | 0.76 | 0.68 | 0.60 | 0.56 | 0.52 | 0.40 | 0.16 | 0.28 | 0.22 | 0.08 | 0.06 |
| IAM | 0.58 | 0.62 | 0.58 | 0.48 | 0.53 | 0.62 | 0.73 | 0.89 | 1.10 | 1.09 | 1.20 | 1.16 | 1.17 | 1.40 | 1.56 | 1.33 | 1.16 | 1.05 | 1.08 | 1.16 | 1.00 |
| LAM | 0.27 | 0.40 | 0.48 | 0.44 | 0.46 | 0.43 | 0.40 | 0.39 | 0.40 | 0.39 | 0.47 | 0.47 | 0.44 | 0.39 | 0.53 | 0.48 | 0.47 | 0.46 | 0.48 | 0.50 | 0.50 |
| Lincosamides | 0.01 | 0.01 | 0.01 | 0.01 | 0.01 | 0.01 | 0.01 | 0.01 | 0.01 | 0.02 | 0.02 | 0.03 | 0.03 | 0.04 | 0.04 | 0.04 | 0.05 | 0.05 | 0.05 | 0.06 | 0.06 |
| Streptogramins | **-** | **-** | **-** | **-** | **-** | **-** | **-** | **-** | **-** | **-** | **-** | **-** | **-** | **-** | **-** | **-** | **-** | **-** | **-** | **-** | **-** |
| **Estonia** | **-** | **-** | **-** | **-** | ***1.45*** | **1.16** | **1.06** | **1.39** | **1.71** | **1.85** | **2.26** | **2.25** | **2.09** | **2.17** | **2.49** | **2.46** | **2.48** | **2.40** | **2.45** | **2.27** | **2.26** |
| SAM | **-** | **-** | **-** | **-** | *0.96* | 0.69 | 0.41 | 0.33 | 0.21 | 0.18 | 0.16 | 0.12 | 0.04 | 0.01 | 0.01 | 0.01 | 0.01 | 0.01 | 0.01 | <0.01 | **-** |
| IAM | **-** | **-** | **-** | **-** | *0.29* | 0.22 | 0.31 | 0.66 | 1.13 | 1.29 | 1.67 | 1.69 | 1.64 | 1.71 | 1.89 | 1.73 | 1.74 | 1.67 | 1.70 | 1.55 | 1.57 |
| LAM | **-** | **-** | **-** | **-** | *0.14* | 0.19 | 0.27 | 0.32 | 0.29 | 0.28 | 0.31 | 0.33 | 0.30 | 0.35 | 0.47 | 0.57 | 0.57 | 0.54 | 0.56 | 0.55 | 0.54 |
| Lincosamides | **-** | **-** | **-** | **-** | *0.07* | 0.07 | 0.07 | 0.07 | 0.09 | 0.09 | 0.12 | 0.12 | 0.11 | 0.10 | 0.12 | 0.15 | 0.17 | 0.17 | 0.18 | 0.18 | 0.15 |
| Streptogramins | **-** | **-** | **-** | **-** | ***-*** | **-** | **-** | **-** | **-** | **-** | **-** | **-** | **-** | **-** | **-** | **-** | **-** | **-** | **-** | **-** | **-** |
| **Finland** | **1.77** | **1.79** | **1.92** | **2.05** | **2.24** | **2.12** | **2.38** | **1.88** | **1.86** | **1.63** | **1.65** | **1.43** | **1.46** | **1.47** | **1.82** | **1.55** | **1.28** | **1.17** | **1.03** | **0.91** | **0.75** |
| SAM | 0.24 | 0.20 | 0.18 | 0.16 | 0.15 | 0.12 | 0.11 | 0.10 | 0.10 | 0.09 | 0.09 | 0.09 | 0.09 | 0.08 | 0.08 | 0.08 | 0.07 | 0.06 | 0.05 | 0.01 | **-** |
| IAM | 0.72 | 0.77 | 0.86 | 1.02 | 1.17 | 1.08 | 1.28 | 1.00 | 0.99 | 0.80 | 0.76 | 0.60 | 0.60 | 0.59 | 0.89 | 0.68 | 0.51 | 0.44 | 0.35 | 0.31 | 0.22 |
| LAM | 0.68 | 0.67 | 0.72 | 0.71 | 0.76 | 0.73 | 0.79 | 0.59 | 0.57 | 0.53 | 0.58 | 0.52 | 0.55 | 0.55 | 0.61 | 0.55 | 0.46 | 0.43 | 0.40 | 0.36 | 0.31 |
| Lincosamides | 0.13 | 0.15 | 0.15 | 0.16 | 0.17 | 0.19 | 0.19 | 0.19 | 0.20 | 0.21 | 0.22 | 0.23 | 0.23 | 0.24 | 0.24 | 0.25 | 0.24 | 0.24 | 0.23 | 0.23 | 0.22 |
| Streptogramins | **-** | **-** | **-** | **-** | **-** | **-** | **-** | **-** | **-** | **-** | **-** | **-** | **-** | **-** | **-** | **-** | **-** | **-** | **-** | **-** | **-** |
| **France** | **5.24** | **5.54** | **5.67** | **6.01** | **6.06** | **5.28** | **4.83** | **4.31** | **4.54** | **3.93** | **4.09** | **4.15** | **4.15** | **3.75** | **3.83** | **3.70** | **3.50** | **3.01** | **3.23** | **2.97** | **3.04** |
| SAM | 0.75 | 0.72 | 0.60 | 0.64 | 0.60 | 0.38 | 0.31 | 0.32 | 0.25 | 0.23 | 0.24 | 0.23 | 0.21 | 0.20 | 0.18 | 0.17 | 0.13 | 0.19 | 0.18 | 0.15 | 0.14 |
| IAM | 3.40 | 3.60 | 3.64 | 3.83 | 3.71 | 3.23 | 2.93 | 2.47 | 2.83 | 2.29 | 2.35 | 2.45 | 2.35 | 1.92 | 1.89 | 1.81 | 1.66 | 1.39 | 1.36 | 1.25 | 1.10 |
| LAM | 0.46 | 0.53 | 0.66 | 0.72 | 0.87 | 0.80 | 0.75 | 0.68 | 0.57 | 0.53 | 0.55 | 0.47 | 0.50 | 0.51 | 0.60 | 0.60 | 0.62 | 0.54 | 0.67 | 0.62 | 0.70 |
| Lincosamides | 0.03 | 0.04 | 0.03 | 0.03 | 0.04 | 0.03 | 0.03 | 0.03 | 0.04 | 0.04 | 0.04 | 0.04 | 0.05 | 0.05 | 0.05 | 0.06 | 0.07 | 0.08 | 0.08 | 0.09 | 0.10 |
| Streptogramins | 0.60 | 0.64 | 0.74 | 0.79 | 0.85 | 0.83 | 0.82 | 0.81 | 0.84 | 0.83 | 0.91 | 0.97 | 1.04 | 1.07 | 1.12 | 1.07 | 1.02 | 0.82 | 0.94 | 0.86 | 1.00 |
| Country, community consumption of macrolides, lincosamides and streptogramins (J01F); SAM, consumption of short-acting macrolides; IAM, consumption of intermediate-acting macrolides; LAM, consumption of  long-acting macrolides; **-**, no consumption reported; Numbers reported in *italic* are total care data, i.e. community and hospital sector combined; ^a^Data for Romania have a coverage in 2009 limited to 30-40%; ^b^Data for Spain include private prescriptions from 2016 onwards. | | | | | | | | | | | | | | | | | | | | | |
| **Germany** | **2.43** | **2.46** | **2.54** | **2.49** | **2.30** | **2.26** | **2.30** | **2.12** | **2.62** | **2.23** | **2.41** | **2.40** | **2.51** | **2.35** | **2.29** | **2.68** | **2.80** | **2.48** | **2.40** | **2.27** | **2.14** |
| SAM | 0.60 | 0.57 | 0.56 | 0.53 | 0.43 | 0.40 | 0.36 | 0.31 | 0.34 | 0.34 | 0.33 | 0.28 | 0.27 | 0.24 | 0.23 | 0.21 | 0.19 | 0.16 | 0.13 | 0.13 | 0.11 |
| IAM | 1.35 | 1.38 | 1.41 | 1.32 | 1.32 | 1.31 | 1.36 | 1.28 | 1.67 | 1.34 | 1.41 | 1.39 | 1.46 | 1.33 | 1.28 | 1.19 | 1.28 | 1.08 | 1.06 | 0.94 | 0.86 |
| LAM | 0.33 | 0.36 | 0.37 | 0.38 | 0.32 | 0.29 | 0.30 | 0.30 | 0.35 | 0.32 | 0.40 | 0.46 | 0.52 | 0.51 | 0.52 | 0.52 | 0.57 | 0.53 | 0.56 | 0.55 | 0.54 |
| Lincosamides | 0.14 | 0.16 | 0.21 | 0.26 | 0.22 | 0.25 | 0.28 | 0.24 | 0.27 | 0.23 | 0.27 | 0.27 | 0.25 | 0.26 | 0.26 | 0.76 | 0.77 | 0.72 | 0.64 | 0.65 | 0.62 |
| Streptogramins | **-** | **-** | **-** | **-** | **-** | **-** | **-** | **-** | **-** | **-** | **-** | **-** | **-** | **-** | **-** | **-** | **-** | **-** | **-** | **-** | **-** |
| **Greece** | **4.16** | **5.37** | **6.28** | **6.79** | **6.92** | **7.79** | **9.32** | ***9.77*** | ***10.08*** | ***10.46*** | ***12.01*** | ***11.60*** | **11.56** | ***9.01*** | **9.54** | **7.81** | **7.22** | **7.88** | **7.50** | **6.07** | **6.98** |
| SAM | 0.94 | 0.88 | 0.68 | 0.97 | 1.07 | 0.87 | 0.82 | *0.65* | *0.58* | *0.27* | *0.21* | *0.17* | 0.08 | *0.13* | 0.09 | 0.06 | 0.06 | <0.01 | <0.01 | <0.01 | <0.01 |
| IAM | 2.85 | 4.05 | 5.30 | 5.58 | 5.49 | 6.50 | 7.99 | *8.39* | *8.48* | *9.36* | *10.40* | *9.71* | 9.85 | *7.42* | 7.88 | 6.42 | 5.88 | 6.63 | 6.06 | 3.85 | 5.39 |
| LAM | 0.24 | 0.28 | 0.15 | 0.05 | 0.07 | 0.09 | 0.22 | *0.49* | *0.73* | *0.81* | *1.11* | *1.40* | 1.31 | *1.16* | 1.32 | 1.10 | 1.00 | 0.94 | 1.09 | 1.90 | 1.25 |
| Lincosamides | 0.14 | 0.16 | 0.16 | 0.19 | 0.29 | 0.33 | 0.29 | *0.25* | *0.29* | *0.02* | *0.28* | *0.33* | 0.31 | *0.30* | 0.26 | 0.23 | 0.28 | 0.31 | 0.35 | 0.33 | 0.35 |
| Streptogramins | **-** | **-** | **-** | **-** | **-** | <0.01 | **-** | ***-*** | *<0.01* | *<0.01* | *<0.01* | *<0.01* | ***-*** | ***-*** | **-** | **-** | **-** | **-** | **-** | **-** | **-** |
| **Hungary** | **-** | **2.80** | **4.14** | **3.38** | **3.16** | **2.78** | **3.06** | **3.09** | **3.90** | **3.46** | **2.89** | **3.07** | **3.00** | **3.00** | **3.05** | **2.73** | **2.82** | **3.13** | **3.30** | **2.82** | **2.80** |
| SAM | **-** | 0.49 | 0.57 | 0.44 | 0.40 | 0.29 | 0.20 | 0.15 | 0.11 | 0.08 | 0.06 | 0.04 | 0.05 | 0.05 | 0.04 | 0.03 | 0.03 | 0.03 | 0.02 | 0.01 | 0.01 |
| IAM | **-** | 1.58 | 2.61 | 2.04 | 1.89 | 1.62 | 1.91 | 1.88 | 2.51 | 2.10 | 1.67 | 1.72 | 1.61 | 1.50 | 1.47 | 1.16 | 1.09 | 1.18 | 1.18 | 0.94 | 0.87 |
| LAM | **-** | 0.37 | 0.53 | 0.47 | 0.40 | 0.31 | 0.33 | 0.38 | 0.58 | 0.63 | 0.48 | 0.63 | 0.73 | 0.88 | 0.96 | 0.96 | 1.11 | 1.31 | 1.50 | 1.28 | 1.36 |
| Lincosamides | **-** | 0.37 | 0.44 | 0.44 | 0.48 | 0.56 | 0.62 | 0.68 | 0.70 | 0.65 | 0.67 | 0.68 | 0.60 | 0.57 | 0.56 | 0.58 | 0.59 | 0.61 | 0.60 | 0.59 | 0.56 |
| Streptogramins | **-** | **-** | **-** | **-** | **-** | **-** | **-** | **-** | **-** | **-** | **-** | **-** | **-** | **-** | **-** | **-** | **-** | **-** | **-** | **-** | **-** |
| **Iceland** | ***1.83*** | ***1.89*** | ***1.77*** | ***1.57*** | ***1.52*** | ***1.55*** | ***0.82*** | ***1.68*** | ***1.83*** | **1.60** | **1.66** | **1.61** | **1.15** | ***1.56*** | ***1.58*** | ***1.66*** | ***1.71*** | **1.56** | **1.71** | **1.69** | **1.61** |
| SAM | *1.55* | *1.48* | *1.34* | *1.07* | *0.96* | *0.88* | *0.03* | *0.77* | *0.76* | 0.54 | 0.52 | 0.42 | 0.28 | *0.42* | *0.28* | *0.34* | *0.24* | 0.25 | 0.19 | 0.19 | 0.19 |
| IAM | *0.11* | *0.12* | *0.16* | *0.18* | *0.20* | *0.22* | *0.29* | *0.30* | *0.35* | 0.34 | 0.39 | 0.39 | 0.37 | *0.31* | *0.36* | *0.33* | *0.28* | 0.24 | 0.26 | 0.23 | 0.24 |
| LAM | *0.14* | *0.26* | *0.24* | *0.28* | *0.32* | *0.39* | *0.44* | *0.53* | *0.63* | 0.63 | 0.66 | 0.69 | 0.43 | *0.66* | *0.76* | *0.79* | *1.01* | 0.95 | 1.12 | 1.12 | 1.00 |
| Lincosamides | *0.04* | *0.04* | *0.03* | *0.04* | *0.05* | *0.06* | *0.06* | *0.07* | *0.10* | 0.08 | 0.09 | 0.10 | 0.07 | *0.16* | *0.17* | *0.20* | *0.18* | 0.13 | 0.14 | 0.15 | 0.18 |
| Streptogramins | ***-*** | ***-*** | ***-*** | ***-*** | ***-*** | ***-*** | ***-*** | ***-*** | ***-*** | **-** | **-** | **-** | **-** | ***-*** | ***-*** | ***-*** | ***-*** | **-** | **-** | **-** | **-** |
| **Ireland** | **-** | **1.94** | **2.17** | **2.13** | **2.30** | **2.45** | **2.80** | **2.87** | **3.12** | **3.53** | **4.13** | **4.12** | **3.79** | **3.66** | **4.17** | **4.16** | **4.38** | **4.15** | **4.20** | **4.38** | **4.19** |
| SAM | **-** | 0.92 | 0.91 | 0.82 | 0.76 | 0.69 | 0.73 | 0.68 | 0.67 | 0.78 | 0.83 | 0.73 | 0.65 | 0.63 | 0.64 | 0.59 | 0.79 | 0.73 | 0.33 | 0.29 | 0.29 |
| IAM | **-** | 0.97 | 1.20 | 1.25 | 1.46 | 1.65 | 1.95 | 2.05 | 2.28 | 2.56 | 3.07 | 3.11 | 2.85 | 2.70 | 3.08 | 2.99 | 2.90 | 2.69 | 3.02 | 3.14 | 2.88 |
| LAM | **-** | 0.04 | 0.06 | 0.06 | 0.07 | 0.09 | 0.10 | 0.12 | 0.15 | 0.17 | 0.21 | 0.24 | 0.25 | 0.30 | 0.41 | 0.53 | 0.62 | 0.66 | 0.78 | 0.89 | 0.94 |
| Lincosamides | **-** | 0.01 | 0.01 | 0.01 | 0.01 | 0.01 | 0.02 | 0.02 | 0.02 | 0.02 | 0.03 | 0.04 | 0.04 | 0.04 | 0.04 | 0.05 | 0.06 | 0.06 | 0.07 | 0.07 | 0.08 |
| Streptogramins | **-** | **-** | **-** | **-** | **-** | **-** | **-** | **-** | **-** | **-** | **-** | **-** | **-** | **-** | **-** | **-** | **-** | **-** | **-** | **-** | **-** |
| Country, community consumption of macrolides, lincosamides and streptogramins (J01F); SAM, consumption of short-acting macrolides; IAM, consumption of intermediate-acting macrolides; LAM, consumption of  long-acting macrolides; **-**, no consumption reported; Numbers reported in *italic* are total care data, i.e. community and hospital sector combined; ^a^Data for Romania have a coverage in 2009 limited to 30-40%; ^b^Data for Spain include private prescriptions from 2016 onwards. | | | | | | | | | | | | | | | | | | | | | |
| **Italy** | **-** | **-** | **5.14** | **5.05** | **5.20** | **5.06** | **4.99** | **4.77** | **4.98** | **4.86** | **4.91** | **5.29** | **5.33** | **5.14** | **4.98** | **4.68** | **4.80** | **4.66** | **4.61** | **4.34** | **3.75** |
| SAM | **-** | **-** | 0.91 | 0.83 | 0.85 | 0.73 | 0.56 | 0.45 | 0.42 | 0.40 | 0.37 | 0.38 | 0.28 | 0.23 | 0.18 | 0.16 | 0.15 | 0.14 | 0.11 | 0.11 | 0.07 |
| IAM | **-** | **-** | 3.08 | 3.07 | 3.03 | 3.09 | 3.17 | 3.07 | 3.25 | 3.16 | 3.19 | 3.51 | 3.59 | 3.40 | 3.26 | 3.03 | 3.05 | 2.97 | 2.89 | 2.70 | 2.27 |
| LAM | **-** | **-** | 1.05 | 1.06 | 1.16 | 1.15 | 1.19 | 1.18 | 1.25 | 1.24 | 1.29 | 1.33 | 1.39 | 1.45 | 1.50 | 1.46 | 1.56 | 1.51 | 1.57 | 1.50 | 1.38 |
| Lincosamides | **-** | **-** | 0.09 | 0.09 | 0.15 | 0.10 | 0.08 | 0.07 | 0.07 | 0.07 | 0.07 | 0.07 | 0.06 | 0.06 | 0.04 | 0.03 | 0.04 | 0.04 | 0.04 | 0.04 | 0.03 |
| Streptogramins | **-** | **-** | **-** | **-** | **-** | **-** | **-** | **-** | **-** | **-** | **-** | **-** | **-** | **-** | **-** | **-** | **-** | **-** | **-** | **-** | **-** |
| **Latvia** | **-** | **-** | **-** | **-** | **-** | **0.75** | **-** | **0.94** | **1.10** | **1.05** | **1.34** | **0.99** | **0.91** | **1.13** | **1.38** | **1.53** | **1.73** | **1.61** | **1.83** | **1.81** | **1.97** |
| SAM | **-** | **-** | **-** | **-** | **-** | 0.49 | **-** | 0.45 | 0.41 | 0.31 | 0.35 | 0.17 | 0.11 | 0.17 | 0.16 | 0.14 | 0.11 | 0.08 | 0.07 | 0.06 | 0.06 |
| IAM | **-** | **-** | **-** | **-** | **-** | 0.16 | **-** | 0.33 | 0.55 | 0.52 | 0.70 | 0.72 | 0.71 | 0.61 | 0.78 | 0.90 | 0.97 | 0.92 | 1.10 | 1.12 | 1.21 |
| LAM | **-** | **-** | **-** | **-** | **-** | 0.04 | **-** | 0.10 | 0.06 | 0.14 | 0.18 | **-** | **-** | 0.25 | 0.33 | 0.37 | 0.53 | 0.48 | 0.53 | 0.50 | 0.57 |
| Lincosamides | **-** | **-** | **-** | **-** | **-** | 0.06 | **-** | 0.07 | 0.08 | 0.09 | 0.11 | 0.10 | 0.09 | 0.10 | 0.11 | 0.12 | 0.12 | 0.12 | 0.12 | 0.14 | 0.13 |
| Streptogramins | **-** | **-** | **-** | **-** | **-** | **-** | **-** | **-** | **-** | **-** | **-** | **-** | **-** | **-** | **-** | **-** | **-** | **-** | **-** | **-** | **-** |
| **Lithuania** | **-** | **-** | **-** | **-** | **-** | **-** | **-** | **-** | **-** | ***1.11*** | ***1.92*** | ***2.04*** | ***1.93*** | ***1.67*** | ***1.91*** | **1.89** | **2.38** | **1.87** | **1.92** | **1.97** | **2.07** |
| SAM | **-** | **-** | **-** | **-** | **-** | **-** | **-** | **-** | **-** | *0.14* | *0.19* | *0.15* | *0.12* | *0.08* | *0.08* | 0.03 | 0.04 | 0.02 | 0.02 | 0.02 | 0.02 |
| IAM | **-** | **-** | **-** | **-** | **-** | **-** | **-** | **-** | **-** | *0.50* | *1.14* | *1.20* | *1.38* | *1.22* | *1.36* | 1.29 | 1.73 | 1.28 | 1.37 | 1.49 | 1.57 |
| LAM | **-** | **-** | **-** | **-** | **-** | **-** | **-** | **-** | **-** | *0.35* | *0.53* | *0.61* | *0.37* | *0.34* | *0.44* | 0.53 | 0.59 | 0.54 | 0.50 | 0.43 | 0.46 |
| Lincosamides | **-** | **-** | **-** | **-** | **-** | **-** | **-** | **-** | **-** | *0.10* | *0.07* | *0.08* | *0.06* | *0.03* | *0.03* | 0.03 | 0.03 | 0.03 | 0.03 | 0.03 | 0.02 |
| Streptogramins | **-** | **-** | **-** | **-** | **-** | **-** | **-** | **-** | **-** | ***-*** | ***-*** | ***-*** | ***-*** | ***-*** | ***-*** | **-** | **-** | **-** | **-** | **-** | **-** |
| **Luxembourg** | **4.84** | **5.05** | **5.25** | **4.98** | **4.91** | **4.42** | **4.39** | **2.76** | **3.07** | **3.38** | **3.79** | **3.80** | **3.87** | **3.73** | **3.90** | **4.02** | **3.95** | **3.66** | **3.64** | **3.85** | **5.50** |
| SAM | 0.86 | 1.02 | 0.90 | 0.80 | 0.78 | 0.69 | 0.77 | 0.55 | 0.59 | 0.48 | 0.53 | 0.46 | 0.43 | 0.38 | 0.39 | 0.31 | 0.23 | 0.21 | 0.19 | 0.20 | 0.38 |
| IAM | 3.09 | 3.19 | 3.38 | 3.06 | 2.88 | 2.45 | 2.34 | 1.16 | 1.31 | 1.83 | 2.07 | 2.10 | 2.11 | 2.06 | 2.14 | 2.26 | 2.24 | 2.04 | 1.96 | 2.01 | 1.42 |
| LAM | 0.59 | 0.51 | 0.66 | 0.79 | 0.90 | 0.92 | 0.95 | 0.67 | 0.79 | 0.73 | 0.83 | 0.85 | 0.92 | 0.89 | 0.95 | 1.01 | 1.04 | 0.98 | 1.04 | 1.17 | 2.47 |
| Lincosamides | 0.29 | 0.31 | 0.29 | 0.31 | 0.31 | 0.34 | 0.33 | 0.38 | 0.38 | 0.34 | 0.37 | 0.38 | 0.41 | 0.39 | 0.42 | 0.43 | 0.44 | 0.44 | 0.46 | 0.47 | 1.23 |
| Streptogramins | 0.01 | 0.01 | 0.02 | 0.02 | 0.03 | 0.02 | <0.01 | **-** | **-** | **-** | **-** | **-** | **-** | **-** | **-** | **-** | **-** | **-** | **-** | **-** | **-** |
| **Malta** | **-** | **-** | **-** | **-** | **-** | **-** | **-** | **-** | **-** | **-** | **3.19** | **3.73** | **3.89** | **3.15** | **3.67** | **3.70** | **3.96** | **3.77** | **4.00** | **3.91** | **4.46** |
| SAM | **-** | **-** | **-** | **-** | **-** | **-** | **-** | **-** | **-** | **-** | 0.33 | 0.24 | 0.26 | 0.29 | 0.20 | 0.24 | 0.21 | 0.16 | 0.15 | 0.15 | 0.11 |
| IAM | **-** | **-** | **-** | **-** | **-** | **-** | **-** | **-** | **-** | **-** | 2.25 | 2.80 | 2.98 | 2.12 | 2.62 | 2.70 | 2.67 | 2.66 | 2.53 | 2.24 | 2.97 |
| LAM | **-** | **-** | **-** | **-** | **-** | **-** | **-** | **-** | **-** | **-** | 0.35 | 0.49 | 0.45 | 0.57 | 0.65 | 0.60 | 0.90 | 0.76 | 1.14 | 1.39 | 1.28 |
| Lincosamides | **-** | **-** | **-** | **-** | **-** | **-** | **-** | **-** | **-** | **-** | 0.26 | 0.20 | 0.21 | 0.18 | 0.20 | 0.16 | 0.18 | 0.18 | 0.19 | 0.13 | 0.09 |
| Streptogramins | **-** | **-** | **-** | **-** | **-** | **-** | **-** | **-** | **-** | **-** | **-** | **-** | **-** | **-** | **-** | **-** | **-** | **-** | **-** | **-** | **-** |
| Country, community consumption of macrolides, lincosamides and streptogramins (J01F); SAM, consumption of short-acting macrolides; IAM, consumption of intermediate-acting macrolides; LAM, consumption of  long-acting macrolides; **-**, no consumption reported; Numbers reported in *italic* are total care data, i.e. community and hospital sector combined; ^a^Data for Romania have a coverage in 2009 limited to 30-40%; ^b^Data for Spain include private prescriptions from 2016 onwards. | | | | | | | | | | | | | | | | | | | | | |
| **Netherlands** | **1.15** | **1.19** | **1.21** | **1.18** | **1.27** | **1.30** | **1.33** | **1.38** | **1.50** | **1.49** | **1.48** | **1.49** | **1.48** | **1.45** | **1.50** | **1.50** | **1.39** | **1.35** | **1.39** | **1.37** | **1.38** |
| SAM | 0.16 | 0.14 | 0.13 | 0.12 | 0.12 | 0.11 | 0.10 | 0.10 | 0.09 | 0.10 | 0.10 | 0.13 | 0.13 | 0.09 | 0.09 | 0.08 | 0.07 | 0.06 | 0.05 | 0.03 | 0.05 |
| IAM | 0.77 | 0.79 | 0.79 | 0.76 | 0.81 | 0.82 | 0.84 | 0.86 | 0.92 | 0.86 | 0.81 | 0.76 | 0.72 | 0.67 | 0.65 | 0.57 | 0.45 | 0.39 | 0.35 | 0.33 | 0.31 |
| LAM | 0.19 | 0.23 | 0.24 | 0.25 | 0.29 | 0.31 | 0.33 | 0.35 | 0.41 | 0.44 | 0.47 | 0.49 | 0.52 | 0.54 | 0.61 | 0.70 | 0.70 | 0.73 | 0.80 | 0.81 | 0.82 |
| Lincosamides | 0.03 | 0.03 | 0.04 | 0.04 | 0.05 | 0.06 | 0.06 | 0.07 | 0.08 | 0.09 | 0.10 | 0.11 | 0.12 | 0.14 | 0.15 | 0.16 | 0.17 | 0.18 | 0.19 | 0.20 | 0.21 |
| Streptogramins | **-** | **-** | **-** | **-** | **-** | **-** | **-** | **-** | **-** | **-** | **-** | **-** | **-** | **-** | **-** | **-** | **-** | **-** | **-** | **-** | **-** |
| **Norway** | **-** | **1.54** | **-** | **-** | **1.75** | **1.92** | **1.85** | **1.80** | **2.04** | **2.00** | **2.06** | **1.89** | **1.68** | **1.79** | **2.00** | **2.00** | **1.69** | **1.47** | **1.31** | **1.15** | **1.01** |
| SAM | **-** | 1.07 | **-** | **-** | 1.13 | 1.19 | 1.08 | 1.02 | 1.15 | 1.13 | 1.11 | 0.98 | 0.84 | 0.85 | 1.01 | 0.95 | 0.75 | 0.68 | 0.60 | 0.54 | 0.48 |
| IAM | **-** | 0.23 | **-** | **-** | 0.30 | 0.35 | 0.36 | 0.35 | 0.38 | 0.37 | 0.40 | 0.35 | 0.29 | 0.32 | 0.34 | 0.37 | 0.28 | 0.22 | 0.18 | 0.13 | 0.11 |
| LAM | **-** | 0.16 | **-** | **-** | 0.21 | 0.24 | 0.26 | 0.28 | 0.32 | 0.32 | 0.36 | 0.35 | 0.34 | 0.38 | 0.40 | 0.43 | 0.37 | 0.31 | 0.29 | 0.26 | 0.23 |
| Lincosamides | **-** | 0.08 | **-** | **-** | 0.12 | 0.13 | 0.15 | 0.16 | 0.20 | 0.18 | 0.19 | 0.21 | 0.21 | 0.23 | 0.24 | 0.25 | 0.28 | 0.26 | 0.24 | 0.22 | 0.19 |
| Streptogramins | **-** | **-** | **-** | **-** | **-** | **-** | **-** | **-** | **-** | **-** | **-** | **-** | **-** | **-** | **-** | **-** | **-** | **-** | **-** | **-** | **-** |
| **Poland** | **-** | **2.06** | **1.97** | **2.39** | **2.64** | **2.40** | **-** | **2.99** | **3.61** | **-** | **4.59** | **3.67** | **3.88** | **3.56** | **3.88** | **3.53** | **3.88** | **3.79** | **4.60** | **4.19** | **4.46** |
| SAM | **-** | 0.36 | 0.34 | 0.41 | 0.44 | 0.35 | **-** | 0.94 | 0.90 | **-** | 1.01 | 0.31 | 0.27 | 0.20 | 0.19 | 0.14 | 0.34 | 0.26 | 0.31 | 0.09 | 0.09 |
| IAM | **-** | 1.04 | 0.91 | 0.99 | 1.23 | 1.14 | **-** | 1.25 | 1.70 | **-** | 2.14 | 1.79 | 1.87 | 1.64 | 1.75 | 1.59 | 1.70 | 1.51 | 1.80 | 1.61 | 1.70 |
| LAM | **-** | 0.14 | 0.17 | 0.31 | 0.38 | 0.35 | **-** | 0.34 | 0.47 | **-** | 0.81 | 0.92 | 1.07 | 1.06 | 1.27 | 1.07 | 1.24 | 1.26 | 1.67 | 1.67 | 1.87 |
| Lincosamides | **-** | 0.52 | 0.55 | 0.69 | 0.59 | 0.56 | **-** | 0.46 | 0.54 | **-** | 0.62 | 0.65 | 0.66 | 0.65 | 0.67 | 0.72 | 0.60 | 0.76 | 0.83 | 0.82 | 0.80 |
| Streptogramins | **-** | **-** | **-** | **-** | **-** | **-** | **-** | **-** | **-** | **-** | **-** | **-** | **-** | **-** | **-** | **-** | **-** | **-** | **-** | **-** | **-** |
| **Portugal** | **2.98** | **3.24** | **3.65** | **3.65** | **3.74** | **3.49** | **3.84** | **3.67** | **4.38** | **3.98** | **-** | **3.88** | **3.83** | **3.41** | **3.39** | **3.21** | **2.75** | **2.79** | **3.06** | **3.09** | **2.44** |
| SAM | 0.79 | 0.69 | 0.63 | 0.52 | 0.42 | 0.35 | 0.29 | 0.26 | 0.24 | 0.28 | **-** | 0.23 | 0.20 | 0.11 | 0.11 | 0.09 | 0.05 | 0.05 | 0.05 | 0.03 | 0.03 |
| IAM | 1.35 | 1.53 | 1.75 | 1.74 | 1.73 | 1.88 | 2.14 | 2.08 | 2.60 | 2.40 | **-** | 2.15 | 2.12 | 1.88 | 1.84 | 1.69 | 1.37 | 1.34 | 1.41 | 1.38 | 1.24 |
| LAM | 0.80 | 0.98 | 1.23 | 1.36 | 1.56 | 1.23 | 1.37 | 1.30 | 1.51 | 1.27 | **-** | 1.47 | 1.47 | 1.39 | 1.40 | 1.39 | 1.29 | 1.36 | 1.56 | 1.63 | 1.13 |
| Lincosamides | 0.03 | 0.03 | 0.03 | 0.03 | 0.03 | 0.03 | 0.03 | 0.03 | 0.03 | 0.03 | **-** | 0.03 | 0.03 | 0.03 | 0.04 | 0.04 | 0.03 | 0.03 | 0.04 | 0.04 | 0.04 |
| Streptogramins | **-** | **-** | **-** | **-** | **-** | **-** | **-** | **-** | **-** | **-** | **-** | **-** | **-** | **-** | **-** | **-** | **-** | **-** | **-** | **-** | **-** |
| **Romania^a^** | **-** | **-** | **-** | **-** | **-** | **-** | **-** | **-** | **-** | **-** | **-** | **-** | ***1.84*** | **-** | ***2.92*** | ***2.67*** | ***2.75*** | ***2.91*** | ***3.18*** | ***2.81*** | ***2.89*** |
| SAM | **-** | **-** | **-** | **-** | **-** | **-** | **-** | **-** | **-** | **-** | **-** | **-** | *0.54* | **-** | *0.43* | *0.33* | *0.26* | *0.23* | *0.25* | *0.17* | *0.10* |
| IAM | **-** | **-** | **-** | **-** | **-** | **-** | **-** | **-** | **-** | **-** | **-** | **-** | *1.10* | **-** | *1.96* | *1.80* | *1.84* | *1.92* | *1.98* | *1.70* | *1.79* |
| LAM | **-** | **-** | **-** | **-** | **-** | **-** | **-** | **-** | **-** | **-** | **-** | **-** | *0.19* | **-** | *0.46* | *0.44* | *0.53* | *0.62* | *0.81* | *0.79* | *0.82* |
| Lincosamides | **-** | **-** | **-** | **-** | **-** | **-** | **-** | **-** | **-** | **-** | **-** | **-** | *<0.01* | **-** | *0.07* | *0.10* | *0.13* | *0.14* | *0.15* | *0.16* | *0.18* |
| Streptogramins | **-** | **-** | **-** | **-** | **-** | **-** | **-** | **-** | **-** | **-** | **-** | **-** | *-* | **-** | ***-*** | ***-*** | ***-*** | ***-*** | ***-*** | ***-*** | ***-*** |
| Country, community consumption of macrolides, lincosamides and streptogramins (J01F); SAM, consumption of short-acting macrolides; IAM, consumption of intermediate-acting macrolides; LAM, consumption of  long-acting macrolides; **-**, no consumption reported; Numbers reported in *italic* are total care data, i.e. community and hospital sector combined; ^a^Data for Romania have a coverage in 2009 limited to 30-40%; ^b^Data for Spain include private prescriptions from 2016 onwards. | | | | | | | | | | | | | | | | | | | | | |
| **Slovakia** | **-** | **-** | **3.42** | **3.09** | **3.43** | **3.51** | **3.82** | **3.32** | **3.94** | **4.96** | **6.29** | **5.91** | **6.09** | **-** | ***5.76*** | **4.91** | **5.89** | **5.56** | **6.20** | **5.44** | **-** |
| SAM | **-** | **-** | 0.68 | 0.54 | 0.57 | 0.38 | 0.32 | 0.28 | 0.36 | 0.34 | 0.34 | 0.30 | 0.30 | **-** | *0.22* | 0.19 | 0.21 | 0.18 | 0.19 | 0.18 | **-** |
| IAM | **-** | **-** | 2.16 | 1.87 | 2.04 | 2.26 | 2.60 | 2.19 | 2.41 | 3.03 | 3.56 | 3.14 | 3.14 | **-** | *2.80* | 2.34 | 2.83 | 2.67 | 2.99 | 2.49 | **-** |
| LAM | **-** | **-** | 0.48 | 0.58 | 0.70 | 0.76 | 0.78 | 0.67 | 0.98 | 1.35 | 2.10 | 2.11 | 2.24 | **-** | *2.33* | 2.01 | 2.42 | 2.27 | 2.56 | 2.29 | **-** |
| Lincosamides | **-** | **-** | 0.11 | 0.11 | 0.12 | 0.11 | 0.12 | 0.17 | 0.19 | 0.23 | 0.28 | 0.35 | 0.40 | **-** | *0.42* | 0.37 | 0.43 | 0.44 | 0.47 | 0.49 | **-** |
| Streptogramins | **-** | **-** | **-** | **-** | **-** | **-** | **-** | **-** | **-** | **-** | **-** | **-** | **-** | **-** | ***-*** | **-** | **-** | **-** | **-** | **-** | **-** |
| **Slovenia** | **2.96** | **3.63** | **3.91** | **3.68** | **3.29** | **2.97** | **3.16** | **3.20** | **3.22** | **2.50** | **2.63** | **2.46** | **2.32** | **2.09** | **1.95** | **1.79** | **1.78** | **1.76** | **1.85** | **1.59** | **1.68** |
| SAM | 0.64 | 0.59 | 0.60 | 0.48 | 0.38 | 0.30 | 0.29 | 0.30 | 0.25 | 0.19 | 0.18 | 0.14 | 0.12 | 0.10 | 0.09 | 0.08 | 0.09 | 0.11 | 0.10 | 0.08 | 0.07 |
| IAM | 0.81 | 1.55 | 1.73 | 1.72 | 1.46 | 1.27 | 1.41 | 1.42 | 1.44 | 1.01 | 1.00 | 0.84 | 0.78 | 0.70 | 0.62 | 0.57 | 0.52 | 0.51 | 0.54 | 0.45 | 0.57 |
| LAM | 1.41 | 1.39 | 1.47 | 1.38 | 1.34 | 1.25 | 1.28 | 1.28 | 1.36 | 1.11 | 1.24 | 1.23 | 1.20 | 1.07 | 1.02 | 0.93 | 0.96 | 0.92 | 0.99 | 0.84 | 0.82 |
| Lincosamides | 0.10 | 0.10 | 0.10 | 0.10 | 0.11 | 0.17 | 0.18 | 0.20 | 0.17 | 0.19 | 0.22 | 0.24 | 0.21 | 0.21 | 0.22 | 0.22 | 0.22 | 0.22 | 0.22 | 0.23 | 0.22 |
| Streptogramins | **-** | **-** | **-** | **-** | **-** | **-** | **-** | **-** | **-** | **-** | **-** | **-** | **-** | **-** | **-** | **-** | **-** | **-** | **-** | **-** | **-** |
| **Spain^b^** | **3.30** | **3.40** | **3.30** | **3.14** | **2.97** | **2.88** | **2.86** | **2.44** | **2.32** | **2.01** | **2.06** | **1.92** | **1.90** | **1.96** | **2.06** | **1.88** | **1.94** | **2.04** | **2.25** | **3.19** | **3.07** |
| SAM | 0.89 | 0.79 | 0.67 | 0.57 | 0.45 | 0.39 | 0.33 | 0.26 | 0.22 | 0.19 | 0.18 | 0.16 | 0.15 | 0.14 | 0.13 | 0.11 | 0.10 | 0.09 | 0.09 | 0.15 | 0.13 |
| IAM | 1.78 | 1.90 | 1.76 | 1.68 | 1.64 | 1.58 | 1.58 | 1.31 | 1.22 | 0.97 | 0.94 | 0.83 | 0.78 | 0.75 | 0.74 | 0.62 | 0.60 | 0.62 | 0.63 | 0.79 | 0.74 |
| LAM | 0.55 | 0.64 | 0.78 | 0.82 | 0.82 | 0.85 | 0.89 | 0.80 | 0.82 | 0.79 | 0.88 | 0.86 | 0.90 | 0.99 | 1.10 | 1.07 | 1.15 | 1.23 | 1.42 | 2.05 | 2.03 |
| Lincosamides | 0.08 | 0.07 | 0.08 | 0.07 | 0.06 | 0.06 | 0.06 | 0.06 | 0.06 | 0.06 | 0.06 | 0.07 | 0.07 | 0.08 | 0.08 | 0.08 | 0.09 | 0.10 | 0.10 | 0.21 | 0.17 |
| Streptogramins | **-** | **-** | **-** | **-** | **-** | **-** | **-** | **-** | **-** | **-** | **-** | **-** | **-** | **-** | **-** | **-** | **-** | **-** | **-** | **-** | **-** |
| **Sweden** | **0.97** | **1.02** | **0.98** | **0.92** | **0.97** | **0.89** | **0.84** | **0.82** | **0.87** | **0.89** | **0.89** | **0.45** | **0.76** | **0.75** | **0.61** | **0.63** | **0.62** | **0.61** | **0.60** | **0.54** | **0.54** |
| SAM | 0.56 | 0.61 | 0.58 | 0.56 | 0.60 | 0.52 | 0.45 | 0.43 | 0.46 | 0.46 | 0.45 | **-** | 0.32 | 0.31 | 0.15 | 0.16 | 0.14 | 0.15 | 0.14 | 0.08 | 0.08 |
| IAM | 0.22 | 0.21 | 0.17 | 0.12 | 0.11 | 0.10 | 0.08 | 0.08 | 0.08 | 0.08 | 0.07 | 0.07 | 0.06 | 0.06 | 0.06 | 0.06 | 0.06 | 0.05 | 0.05 | 0.05 | 0.05 |
| LAM | 0.02 | 0.03 | 0.03 | 0.03 | 0.03 | 0.03 | 0.03 | 0.04 | 0.05 | 0.05 | 0.05 | 0.06 | 0.06 | 0.06 | 0.07 | 0.09 | 0.10 | 0.09 | 0.09 | 0.09 | 0.10 |
| Lincosamides | 0.16 | 0.18 | 0.19 | 0.21 | 0.23 | 0.24 | 0.27 | 0.27 | 0.28 | 0.31 | 0.31 | 0.32 | 0.32 | 0.32 | 0.33 | 0.32 | 0.32 | 0.32 | 0.32 | 0.32 | 0.31 |
| Streptogramins | **-** | **-** | **-** | **-** | **-** | **-** | **-** | **-** | **-** | **-** | **-** | **-** | **-** | **-** | **-** | **-** | **-** | **-** | **-** | **-** | **-** |
| **United Kingdom** | **2.85** | **2.72** | **2.46** | **2.31** | **2.34** | **2.30** | **2.33** | **2.24** | **2.29** | **2.20** | **2.38** | **2.47** | **2.51** | **2.73** | **2.81** | **3.10** | **3.20** | **3.24** | **3.10** | **3.02** | **2.90** |
| SAM | 2.31 | 2.19 | 1.99 | 1.87 | 1.86 | 1.80 | 1.79 | 1.69 | 1.67 | 1.55 | 1.59 | 1.57 | 1.45 | 1.39 | 1.30 | 1.26 | 1.14 | 1.03 | 0.87 | 0.76 | 0.65 |
| IAM | 0.49 | 0.49 | 0.43 | 0.41 | 0.44 | 0.45 | 0.48 | 0.48 | 0.54 | 0.55 | 0.65 | 0.75 | 0.88 | 1.12 | 1.25 | 1.53 | 1.64 | 1.74 | 1.72 | 1.73 | 1.70 |
| LAM | 0.04 | 0.03 | 0.03 | 0.03 | 0.03 | 0.03 | 0.04 | 0.05 | 0.06 | 0.07 | 0.11 | 0.13 | 0.15 | 0.19 | 0.23 | 0.28 | 0.37 | 0.41 | 0.45 | 0.46 | 0.49 |
| Lincosamides | 0.01 | 0.01 | 0.01 | 0.01 | 0.01 | 0.01 | 0.02 | 0.02 | 0.02 | 0.02 | 0.02 | 0.03 | 0.03 | 0.03 | 0.03 | 0.03 | 0.06 | 0.06 | 0.06 | 0.06 | 0.07 |
| Streptogramins | **-** | **-** | **-** | **-** | **-** | **-** | **-** | **-** | **-** | **-** | **-** | **-** | **-** | **-** | **-** | **-** | **-** | **-** | **-** | **-** | **-** |

Country, community consumption of macrolides, lincosamides and streptogramins (J01F); SAM, consumption of short-acting macrolides; IAM, consumption of intermediate-acting macrolides; LAM, consumption of long-acting macrolides; **-**, no consumption reported; Numbers reported in *italic* are total care data, i.e. community and hospital sector combined; ^a^Data for Romania have a coverage in 2009 limited to 30-40%; ^b^Data for Spain include private prescriptions from 2016 onwards.

**Table S2. Consumption of macrolides, lincosamides and streptogramins (ATC J01F) in the community, expressed in packages per 1000 inhabitants per day, 23 EU/EEA countries, 2006-2017.**

| **Country** | **2006** | **2007** | **2008** | **2009** | **2010** | **2011** | **2012** | **2013** | **2014** | **2015** | **2016** | **2017** |  |
| --- | --- | --- | --- | --- | --- | --- | --- | --- | --- | --- | --- | --- | --- |
| **Austria** | **-** | **0.58** | **0.58** | **0.60** | **0.55** | **0.52** | **0.50** | **0.56** | **0.47** | **0.48** | **0.43** | **0.44** |  |
| SAM | **-** | 0.01 | 0.01 | 0.01 | <0.01 | <0.01 | <0.01 | <0.01 | <0.01 | <0.01 | <0.01 | <0.01 |  |
| IAM | **-** | 0.32 | 0.31 | 0.32 | 0.28 | 0.26 | 0.23 | 0.25 | 0.20 | 0.20 | 0.17 | 0.16 |  |
| LAM | **-** | 0.13 | 0.13 | 0.14 | 0.13 | 0.13 | 0.13 | 0.16 | 0.14 | 0.15 | 0.14 | 0.16 |  |
| Lincosamides | **-** | 0.13 | 0.13 | 0.13 | 0.14 | 0.13 | 0.13 | 0.15 | 0.13 | 0.12 | 0.12 | 0.12 |  |
| Streptogramins | **-** | **-** | **-** | **-** | **-** | **-** | **-** | **-** | **-** | **-** | **-** | **-** |  |
| **Belgium^a^** | **-** | **0.37** | **0.38** | **0.39** | **0.39** | **0.42** | **0.43** | **0.43** | **0.43** | **0.45** | **0.48** | **0.44** |  |
| SAM | **-** | 0.03 | 0.03 | 0.03 | 0.03 | 0.03 | 0.02 | 0.02 | 0.02 | 0.02 | 0.01 | 0.01 |  |
| IAM | **-** | 0.17 | 0.16 | 0.15 | 0.14 | 0.14 | 0.14 | 0.13 | 0.12 | 0.12 | 0.12 | 0.10 |  |
| LAM | **-** | 0.11 | 0.12 | 0.14 | 0.14 | 0.17 | 0.19 | 0.20 | 0.21 | 0.23 | 0.25 | 0.24 |  |
| Lincosamides | **-** | 0.06 | 0.07 | 0.07 | 0.08 | 0.08 | 0.09 | 0.08 | 0.08 | 0.08 | 0.11 | 0.10 |  |
| Streptogramins | **-** | **-** | **-** | **-** | **-** | **-** | **-** | **-** | **-** | **-** | **-** | **-** |  |
| **Bulgaria** | **0.36** | **0.49** | **0.57** | **0.56** | **0.58** | **0.61** | **0.57** | **0.64** | **0.70** | **0.68** | **0.65** | **0.66** |  |
| SAM | 0.08 | 0.07 | 0.05 | 0.04 | 0.03 | 0.03 | 0.02 | 0.02 | 0.02 | 0.01 | 0.01 | 0.01 |  |
| IAM | 0.10 | 0.18 | 0.23 | 0.23 | 0.23 | 0.25 | 0.22 | 0.24 | 0.26 | 0.25 | 0.22 | 0.23 |  |
| LAM | 0.13 | 0.17 | 0.21 | 0.20 | 0.22 | 0.23 | 0.21 | 0.24 | 0.30 | 0.30 | 0.28 | 0.29 |  |
| Lincosamides | 0.05 | 0.07 | 0.08 | 0.09 | 0.09 | 0.10 | 0.12 | 0.14 | 0.12 | 0.13 | 0.13 | 0.13 |  |
| Streptogramins | **-** | **-** | **-** | **-** | **-** | **-** | **-** | **-** | **-** | **-** | **-** | **-** |  |
| **Croatia** | **-** | **0.62** | **0.61** | **0.62** | **0.53** | **0.52** | **0.53** | **0.51** | **0.54** | **0.57** | **0.49** | **0.50** |  |
| SAM | **-** | 0.02 | 0.02 | 0.02 | 0.01 | 0.01 | 0.01 | 0.01 | 0.01 | 0.01 | <0.01 | <0.01 |  |
| IAM | **-** | 0.14 | 0.14 | 0.13 | 0.13 | 0.11 | 0.10 | 0.09 | 0.08 | 0.09 | 0.08 | 0.08 |  |
| LAM | **-** | 0.38 | 0.37 | 0.41 | 0.36 | 0.33 | 0.36 | 0.35 | 0.37 | 0.40 | 0.33 | 0.33 |  |
| Lincosamides | **-** | 0.07 | 0.07 | 0.07 | 0.03 | 0.07 | 0.07 | 0.07 | 0.08 | 0.08 | 0.08 | 0.08 |  |
| Streptogramins | **-** | **-** | **-** | **-** | **-** | **-** | **-** | **-** | **-** | **-** | **-** | **-** |  |
| **Czechia** | **-** | **0.49** | **-** | **-** | **0.55** | **0.57** | **0.53** | **0.57** | **0.58** | **0.59** | **-** | **-** |  |
| SAM | **-** | 0.06 | **-** | **-** | 0.04 | 0.03 | 0.03 | 0.03 | 0.02 | 0.02 | **-** | **-** |  |
| IAM | **-** | 0.26 | **-** | **-** | 0.24 | 0.26 | 0.25 | 0.27 | 0.27 | 0.26 | **-** | **-** |  |
| LAM | **-** | 0.13 | **-** | **-** | 0.16 | 0.16 | 0.15 | 0.17 | 0.17 | 0.18 | **-** | **-** |  |
| Lincosamides | **-** | 0.04 | **-** | **-** | 0.11 | 0.11 | 0.11 | 0.10 | 0.12 | 0.12 | **-** | **-** |  |
| Streptogramins | **-** |  | **-** | **-** | **-** | **-** | **-** | **-** | **-** | **-** | **-** | **-** |  |
| Country, community consumption of macrolides, lincosamides and streptogramins (J01F); SAM, consumption of short-acting macrolides; IAM, consumption of intermediate-acting macrolides; LAM, consumption of long-acting macrolides; **-**, no consumption reported; Numbers reported in *italic* are total care data, i.e. community and hospital sector combined; ^a^Data for Belgium are slightly overestimated from 2016 onwards (nursing homes counting units versus packages before 2016); ^b^Data for the Netherlands are based on average package size; ^c^Data for Spain include private prescriptions from 2016 onwards. | | | | | | | | | | | | | |
| **Denmark** | **-** | **0.30** | **0.29** | **0.28** | **0.29** | **0.33** | **0.28** | **0.25** | **0.24** | **0.24** | **0.24** | **0.22** |  |
| SAM | **-** | 0.07 | 0.06 | 0.05 | 0.05 | 0.04 | 0.03 | 0.02 | 0.02 | 0.02 | 0.01 | 0.01 |  |
| IAM | **-** | 0.13 | 0.12 | 0.12 | 0.15 | 0.16 | 0.14 | 0.12 | 0.10 | 0.11 | 0.11 | 0.09 |  |
| LAM | **-** | 0.10 | 0.10 | 0.10 | 0.09 | 0.12 | 0.11 | 0.10 | 0.10 | 0.11 | 0.11 | 0.11 |  |
| Lincosamides | **-** | <0.01 | 0.01 | 0.01 | 0.01 | 0.01 | 0.01 | 0.01 | 0.01 | 0.01 | 0.01 | 0.01 |  |
| Streptogramins | **-** | **-** | **-** | **-** | **-** | **-** | **-** | **-** | **-** | **-** | **-** | **-** |  |
| **Estonia** | **0.32** | **0.37** | **0.36** | **0.33** | **0.33** | **0.37** | **0.38** | **0.37** | **0.35** | **0.35** | **0.33** | **0.31** |  |
| SAM | 0.04 | 0.04 | 0.03 | 0.01 | <0.01 | <0.01 | <0.01 | <0.01 | <0.01 | <0.01 | **-** | **-** |  |
| IAM | 0.19 | 0.24 | 0.23 | 0.22 | 0.23 | 0.24 | 0.22 | 0.21 | 0.20 | 0.19 | 0.17 | 0.17 |  |
| LAM | 0.06 | 0.06 | 0.07 | 0.06 | 0.07 | 0.09 | 0.12 | 0.12 | 0.11 | 0.12 | 0.11 | 0.11 |  |
| Lincosamides | 0.03 | 0.03 | 0.03 | 0.03 | 0.03 | 0.03 | 0.04 | 0.04 | 0.04 | 0.04 | 0.04 | 0.03 |  |
| Streptogramins | **-** | **-** | **-** | **-** | **-** | **-** | **-** | **-** | **-** | **-** | **-** | **-** |  |
| **Finland** | **-** | **-** | **0.24** | **0.24** | **0.25** | **0.30** | **0.25** | **0.21** | **0.19** | **0.17** | **0.15** | **0.13** |  |
| SAM | **-** | **-** | <0.01 | <0.01 | <0.01 | <0.01 | <0.01 | <0.01 | <0.01 | <0.01 | <0.01 | **-** |  |
| IAM | **-** | **-** | 0.07 | 0.07 | 0.07 | 0.10 | 0.08 | 0.06 | 0.05 | 0.04 | 0.04 | 0.03 |  |
| LAM | **-** | **-** | 0.13 | 0.13 | 0.14 | 0.15 | 0.13 | 0.11 | 0.10 | 0.09 | 0.08 | 0.07 |  |
| Lincosamides | **-** | **-** | 0.03 | 0.03 | 0.04 | 0.04 | 0.04 | 0.04 | 0.04 | 0.03 | 0.03 | 0.03 |  |
| Streptogramins | **-** | **-** | **-** | **-** | **-** | **-** | **-** | **-** | **-** | **-** | **-** | **-** |  |
| **France** | **-** | **-** | **-** | **-** | **0.74** | **0.76** | **0.74** | **0.70** | **0.61** | **0.66** | **0.60** | **0.63** |  |
| SAM | **-** | **-** | **-** | **-** | 0.04 | 0.04 | 0.04 | 0.03 | 0.04 | 0.04 | 0.03 | 0.03 |  |
| IAM | **-** | **-** | **-** | **-** | 0.31 | 0.31 | 0.30 | 0.27 | 0.23 | 0.22 | 0.20 | 0.18 |  |
| LAM | **-** | **-** | **-** | **-** | 0.10 | 0.11 | 0.12 | 0.13 | 0.11 | 0.14 | 0.13 | 0.14 |  |
| Lincosamides | **-** | **-** | **-** | **-** | 0.01 | 0.01 | 0.02 | 0.02 | 0.02 | 0.02 | 0.02 | 0.02 |  |
| Streptogramins | **-** | **-** | **-** | **-** | 0.27 | 0.28 | 0.27 | 0.26 | 0.21 | 0.24 | 0.22 | 0.25 |  |
| **Greece** | ***1.12*** | ***1.34*** | ***1.30*** | **1.09** | ***1.00*** | **0.89** | **0.74** | **0.72** | **0.74** | **0.75** | **0.72** | **0.72** |  |
| SAM | *0.06* | *0.04* | *0.04* | 0.03 | *0.03* | 0.03 | 0.01 | 0.01 | <0.01 | <0.01 | <0.01 | <0.01 |  |
| IAM | *0.81* | *0.81* | *0.71* | 0.71 | *0.53* | 0.55 | 0.46 | 0.42 | 0.47 | 0.43 | 0.39 | 0.37 |  |
| LAM | *0.18* | *0.25* | *0.28* | 0.24 | *0.21* | 0.21 | 0.18 | 0.18 | 0.17 | 0.21 | 0.22 | 0.23 |  |
| Lincosamides | *0.07* | *0.24* | *0.27* | 0.11 | *0.23* | 0.10 | 0.09 | 0.10 | 0.10 | 0.11 | 0.11 | 0.12 |  |
| Streptogramins | *<0.01* | *<0.01* | *<0.01* | ***-*** | *<0.01* | **-** | **-** | **-** | **-** | **-** | **-** | **-** |  |
| Country, community consumption of macrolides, lincosamides and streptogramins (J01F); SAM, consumption of short-acting macrolides; IAM, consumption of intermediate-acting macrolides; LAM, consumption of long-acting macrolides; **-**, no consumption reported; Numbers reported in *italic* are total care data, i.e. community and hospital sector combined; ^a^Data for Belgium are slightly overestimated from 2016 onwards (nursing homes counting units versus packages before 2016); ^b^Data for the Netherlands are based on average package size; ^c^Data for Spain include private prescriptions from 2016 onwards. | | | | | | | | | | | | | |
| **Iceland** | **-** | **-** | **-** | **-** | ***0.33*** | ***0.34*** | ***0.36*** | ***0.39*** | **0.28** | **0.31** | **0.31** | **0.29** |  |
| SAM | **-** | **-** | **-** | **-** | *0.05* | *0.03* | *0.04* | *0.03* | 0.03 | 0.02 | 0.02 | 0.02 |  |
| IAM | **-** | **-** | **-** | **-** | *0.03* | *0.03* | *0.03* | *0.02* | 0.02 | 0.02 | 0.02 | 0.02 |  |
| LAM | **-** | **-** | **-** | **-** | *0.16* | *0.18* | *0.19* | *0.22* | 0.21 | 0.24 | 0.24 | 0.21 |  |
| Lincosamides | **-** | **-** | **-** | **-** | *0.09* | *0.10* | *0.11* | *0.12* | 0.03 | 0.03 | 0.03 | 0.04 |  |
| Streptogramins | **-** | **-** | **-** | **-** | ***-*** | ***-*** | ***-*** | ***-*** | **-** | **-** | **-** | **-** |  |
| **Ireland** | **-** | **0.49** | **-** | **-** | **0.44** | **0.50** | **0.52** | **0.51** | **0.49** | **0.56** | **0.58** | **0.55** |  |
| SAM | **-** | 0.06 | **-** | **-** | 0.04 | 0.05 | 0.05 | 0.05 | 0.04 | 0.04 | 0.04 | 0.03 |  |
| IAM | **-** | 0.38 | **-** | **-** | 0.31 | 0.35 | 0.33 | 0.31 | 0.28 | 0.33 | 0.33 | 0.30 |  |
| LAM | **-** | 0.05 | **-** | **-** | 0.07 | 0.09 | 0.12 | 0.14 | 0.15 | 0.17 | 0.19 | 0.21 |  |
| Lincosamides | **-** | 0.01 | **-** | **-** | 0.01 | 0.01 | 0.01 | 0.01 | 0.02 | 0.02 | 0.02 | 0.02 |  |
| Streptogramins | **-** | **-** | **-** | **-** | **-** | **-** | **-** | **-** | **-** | **-** | **-** | **-** |  |
| **Italy** | **-** | **-** | **0.84** | **-** | **0.80** | **0.77** | **0.72** | **0.73** | **0.71** | **0.70** | **0.66** | **0.56** |  |
| SAM | **-** | **-** | 0.08 | **-** | 0.06 | 0.05 | 0.04 | 0.04 | 0.04 | 0.03 | 0.03 | 0.02 |  |
| IAM | **-** | **-** | 0.37 | **-** | 0.34 | 0.33 | 0.30 | 0.30 | 0.28 | 0.27 | 0.26 | 0.21 |  |
| LAM | **-** | **-** | 0.26 | **-** | 0.29 | 0.30 | 0.29 | 0.31 | 0.30 | 0.31 | 0.30 | 0.28 |  |
| Lincosamides | **-** | **-** | 0.13 | **-** | 0.11 | 0.10 | 0.09 | 0.08 | 0.08 | 0.08 | 0.07 | 0.05 |  |
| Streptogramins | **-** | **-** | **-** | **-** | **-** | **-** | **-** | **-** | **-** | **-** | **-** | **-** |  |
| **Latvia** | **-** | **-** | **-** | **-** | **0.21** | **0.26** | **0.26** | **0.29** | **0.26** | **0.28** | **0.27** | **0.29** |  |
| SAM | **-** | **-** | **-** | **-** | 0.06 | 0.06 | 0.04 | 0.03 | 0.02 | 0.02 | 0.02 | 0.02 |  |
| IAM | **-** | **-** | **-** | **-** | 0.07 | 0.10 | 0.11 | 0.11 | 0.10 | 0.12 | 0.12 | 0.13 |  |
| LAM | **-** | **-** | **-** | **-** | 0.05 | 0.06 | 0.07 | 0.11 | 0.10 | 0.11 | 0.10 | 0.12 |  |
| Lincosamides | **-** | **-** | **-** | **-** | 0.03 | 0.03 | 0.04 | 0.04 | 0.04 | 0.04 | 0.04 | 0.03 |  |
| Streptogramins | **-** | **-** | **-** | **-** | **-** | **-** | **-** | **-** | **-** | **-** | **-** | **-** |  |
| **Lithuania** | ***-*** | ***0.36*** | ***0.38*** | ***0.33*** | ***0.27*** | ***0.30*** | **0.29** | **0.34** | **0.28** | **0.28** | **0.27** | **0.27** |  |
| SAM | ***-*** | *0.06* | *0.05* | *0.04* | *0.03* | *0.02* | 0.02 | 0.02 | 0.01 | 0.01 | 0.01 | 0.01 |  |
| IAM | ***-*** | *0.16* | *0.16* | *0.18* | *0.16* | *0.17* | 0.16 | 0.20 | 0.15 | 0.15 | 0.16 | 0.16 |  |
| LAM | ***-*** | *0.11* | *0.13* | *0.08* | *0.07* | *0.09* | 0.11 | 0.12 | 0.11 | 0.10 | 0.09 | 0.10 |  |
| Lincosamides | ***-*** | *0.03* | *0.03* | *0.03* | *0.02* | *0.02* | 0.01 | 0.01 | 0.01 | 0.01 | 0.01 | 0.01 |  |
| Streptogramins | ***-*** | ***-*** | ***-*** | ***-*** | ***-*** | ***-*** | **-** | **-** | **-** | **-** | **-** | **-** |  |
| Country, community consumption of macrolides, lincosamides and streptogramins (J01F); SAM, consumption of short-acting macrolides; IAM, consumption of intermediate-acting macrolides; LAM, consumption of long-acting macrolides; **-**, no consumption reported; Numbers reported in *italic* are total care data, i.e. community and hospital sector combvined; ^a^Data for Belgium are slightly overestimated from 2016 onwards (nursing homes counting units versus packages before 2016); ^b^Data for the Netherlands are based on average package size; ^c^Data for Spain include private prescriptions from 2016 onwards. | | | | | | | | | | | | | |
| **Luxembourg** | **0.59** | **0.63** | **0.62** | **0.63** | **0.59** | **0.60** | **0.60** | **0.59** | **0.55** | **0.55** | **0.59** | **-** |  |
| SAM | 0.10 | 0.11 | 0.10 | 0.09 | 0.08 | 0.08 | 0.06 | 0.05 | 0.05 | 0.04 | 0.04 | **-** |  |
| IAM | 0.23 | 0.24 | 0.24 | 0.24 | 0.21 | 0.20 | 0.21 | 0.20 | 0.18 | 0.17 | 0.18 | **-** |  |
| LAM | 0.16 | 0.18 | 0.19 | 0.20 | 0.19 | 0.21 | 0.22 | 0.22 | 0.21 | 0.22 | 0.25 | **-** |  |
| Lincosamides | 0.09 | 0.10 | 0.10 | 0.11 | 0.10 | 0.11 | 0.11 | 0.11 | 0.11 | 0.12 | 0.12 | **-** |  |
| Streptogramins | **-** | **-** | **-** | **-** | **-** | **-** | **-** | **-** | **-** | **-** | **-** | **-** |  |
| **Netherlands^b^** | **-** | **-** | **0.22** | **0.22** | **-** | **-** | **-** | **-** | **-** | **-** | **-** | **-** |  |
| SAM | **-** | **-** | 0.01 | 0.01 | **-** | **-** | **-** | **-** | **-** | **-** | **-** | **-** |  |
| IAM | **-** | **-** | 0.09 | 0.08 | **-** | **-** | **-** | **-** | **-** | **-** | **-** | **-** |  |
| LAM | **-** | **-** | 0.11 | 0.11 | **-** | **-** | **-** | **-** | **-** | **-** | **-** | **-** |  |
| Lincosamides | **-** | **-** | 0.02 | 0.02 | **-** | **-** | **-** | **-** | **-** | **-** | **-** | **-** |  |
| Streptogramins | **-** | **-** | **-** | **-** | **-** | **-** | **-** | **-** | **-** | **-** | **-** | **-** |  |
| **Portugal** | **-** | **-** | **0.54** | **0.53** | **0.49** | **0.51** | **0.49** | **0.42** | **0.44** | **0.49** | **0.50** | **0.46** |  |
| SAM | **-** | **-** | 0.04 | 0.04 | 0.03 | 0.03 | 0.03 | 0.02 | 0.02 | 0.02 | 0.01 | 0.01 |  |
| IAM | **-** | **-** | 0.18 | 0.18 | 0.16 | 0.16 | 0.15 | 0.12 | 0.12 | 0.12 | 0.12 | 0.11 |  |
| LAM | **-** | **-** | 0.30 | 0.30 | 0.29 | 0.29 | 0.29 | 0.27 | 0.28 | 0.33 | 0.34 | 0.32 |  |
| Lincosamides | **-** | **-** | 0.02 | 0.02 | 0.02 | 0.02 | 0.02 | 0.02 | 0.02 | 0.02 | 0.02 | 0.02 |  |
| Streptogramins | **-** | **-** | **-** | **-** | **-** | **-** | **-** | **-** | **-** | **-** | **-** | **-** |  |
| **Slovakia** | **-** | **-** | **-** | **-** | **-** | ***0.93*** | **0.79** | **0.92** | **0.85** | **0.92** | **0.81** | **-** |  |
| SAM | **-** | **-** | **-** | **-** | **-** | *0.07* | 0.06 | 0.07 | 0.06 | 0.06 | 0.06 | **-** |  |
| IAM | **-** | **-** | **-** | **-** | **-** | *0.32* | 0.26 | 0.31 | 0.30 | 0.33 | 0.26 | **-** |  |
| LAM | **-** | **-** | **-** | **-** | **-** | *0.43* | 0.37 | 0.43 | 0.38 | 0.42 | 0.37 | **-** |  |
| Lincosamides | **-** | **-** | **-** | **-** | **-** | *0.11* | 0.10 | 0.11 | 0.11 | 0.12 | 0.13 | **-** |  |
| Streptogramins | **-** | **-** | **-** | **-** | **-** | ***-*** | **-** | **-** | **-** | **-** | **-** | **-** |  |
| **Slovenia** | **-** | **0.46** | **0.44** | **0.41** | **0.37** | **0.35** | **0.32** | **0.33** | **0.33** | **0.34** | **0.29** | **0.29** |  |
| SAM | **-** | 0.04 | 0.03 | 0.03 | 0.02 | 0.02 | 0.02 | 0.02 | 0.02 | 0.02 | 0.02 | 0.02 |  |
| IAM | **-** | 0.09 | 0.08 | 0.07 | 0.06 | 0.05 | 0.05 | 0.04 | 0.04 | 0.04 | 0.03 | 0.04 |  |
| LAM | **-** | 0.27 | 0.27 | 0.26 | 0.23 | 0.22 | 0.20 | 0.21 | 0.21 | 0.22 | 0.18 | 0.18 |  |
| Lincosamides | **-** | 0.06 | 0.07 | 0.06 | 0.06 | 0.06 | 0.06 | 0.06 | 0.06 | 0.06 | 0.06 | 0.06 |  |
| Streptogramins | **-** | **-** | **-** | **-** | **-** | **-** | **-** | **-** | **-** | **-** | **-** | **-** |  |
| Country, community consumption of macrolides, lincosamides and streptogramins (J01F); SAM, consumption of short-acting macrolides; IAM, consumption of intermediate-acting macrolides; LAM, consumption of long-acting macrolides; **-**, no consumption reported; Numbers reported in *italic* are total care data, i.e. community and hospital sector combined; ^a^Data for Belgium are slightly overestimated from 2016 onwards (nursing homes counting units versus packages before 2016); ^b^Data for the Netherlands are based on average package size; ^c^Data for Spain include private prescriptions from 2016 onwards. | | | | | | | | | | | | | |
| **Spain^c^** | **-** | **-** | **-** | **-** | **0.33** | **0.35** | **0.32** | **0.33** | **0.34** | **0.38** | **0.55** | **0.54** |  |
| SAM | **-** | **-** | **-** | **-** | 0.04 | 0.03 | 0.03 | 0.02 | 0.02 | 0.01 | 0.02 | 0.02 |  |
| IAM | **-** | **-** | **-** | **-** | 0.06 | 0.06 | 0.05 | 0.05 | 0.04 | 0.04 | 0.05 | 0.05 |  |
| LAM | **-** | **-** | **-** | **-** | 0.21 | 0.24 | 0.23 | 0.25 | 0.26 | 0.30 | 0.44 | 0.43 |  |
| Lincosamides | **-** | **-** | **-** | **-** | 0.01 | 0.01 | 0.01 | 0.02 | 0.02 | 0.02 | 0.04 | 0.03 |  |
| Streptogramins | **-** | **-** | **-** | **-** | **-** | **-** | **-** | **-** | **-** | **-** | **-** | **-** |  |
| **Sweden** | **-** | **-** | **-** | **0.09** | **0.09** | **0.09** | **0.09** | **0.08** | **0.08** | **0.08** | **0.08** | **0.08** |  |
| SAM | **-** | **-** | **-** | 0.03 | 0.03 | 0.03 | 0.03 | 0.01 | 0.02 | 0.02 | 0.01 | 0.01 |  |
| IAM | **-** | **-** | **-** | <0.01 | <0.01 | <0.01 | <0.01 | <0.01 | <0.01 | <0.01 | <0.01 | <0.01 |  |
| LAM | **-** | **-** | **-** | 0.01 | 0.01 | 0.01 | 0.01 | 0.01 | 0.01 | 0.01 | 0.01 | 0.01 |  |
| Lincosamides | **-** | **-** | **-** | 0.05 | 0.05 | 0.05 | 0.05 | 0.05 | 0.05 | 0.05 | 0.04 | 0.04 |  |
| Streptogramins | **-** | **-** | **-** | **-** | **-** | **-** | **-** | **-** | **-** | **-** | **-** | **-** |  |
| **United Kingdom** | **-** | **-** | **-** | **-** | **-** | **-** | **-** | **-** | **-** | **0.01** | **-** | **-** |  |
| SAM | **-** | **-** | **-** | **-** | **-** | **-** | **-** | **-** | **-** | <0.01 | **-** | **-** |  |
| IAM | **-** | **-** | **-** | **-** | **-** | **-** | **-** | **-** | **-** | 0.01 | **-** | **-** |  |
| LAM | **-** | **-** | **-** | **-** | **-** | **-** | **-** | **-** | **-** | <0.01 | **-** | **-** |  |
| Lincosamides | **-** | **-** | **-** | **-** | **-** | **-** | **-** | **-** | **-** | <0.01 | **-** | **-** |  |
| Streptogramins | **-** | **-** | **-** | **-** | **-** | **-** | **-** | **-** | **-** | - | **-** | **-** |  |

Country, community consumption of macrolides, lincosamides and streptogramins (J01F); SAM, consumption of short-acting macrolides; IAM, consumption of intermediate-acting macrolides; LAM,
consumption of long-acting macrolides; **-**, no consumption reported; Numbers reported in *italic* are total care data, i.e. community and hospital sector combined; ^a^Data for Belgium are slightly overestimated from 2016 onwards (nursing homes counting units versus packages before 2016); ^b^Data for the Netherlands are based on average package size; ^c^Data for Spain include private prescriptions from 2016 onwards.

**
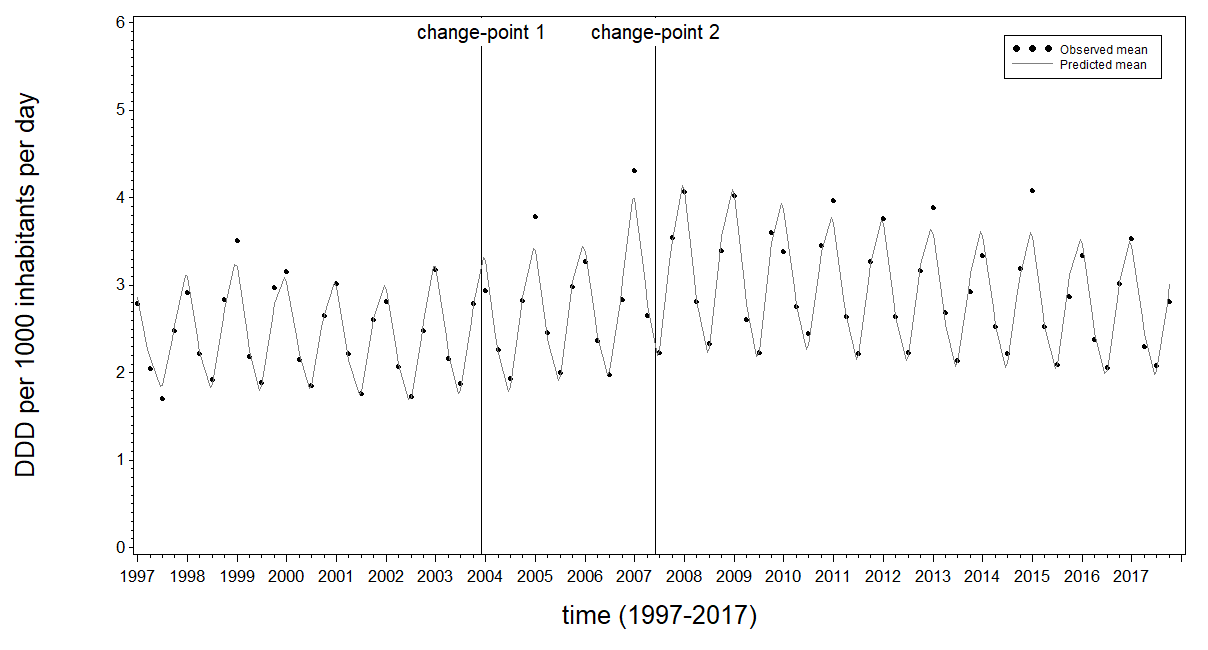
**

**Figure S1. Average of observed (dots) and predicted (solid line) consumption of macrolides, lincosamides and streptogramins (ATC J01F) in the community expressed in DDD (ATC/DDD index 2019) per 1000 inhabitants per day and based on quarterly data, 25 EU/EEA countries, 1997-2017.**


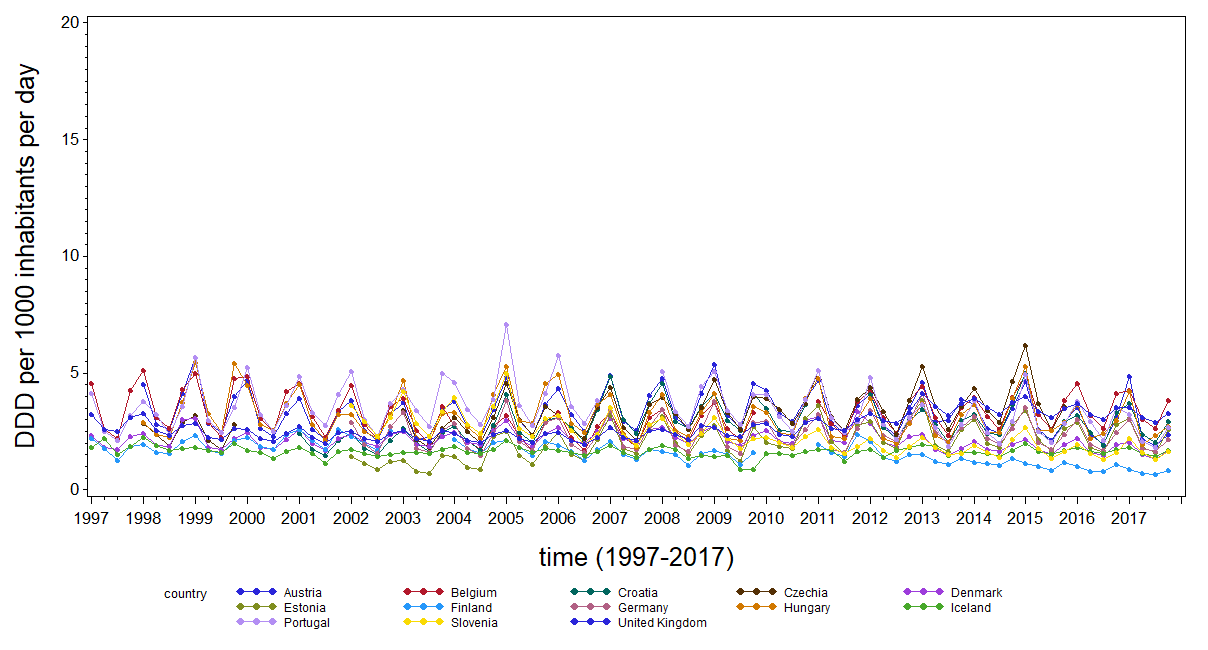


**Figure S2. Seasonal variation in consumption of macrolides, lincosamides and streptogramins (ATC J01F) in the community, expressed in DDD (ATC/DDD index 2019) per 1000 inhabitants per day, 13 EU/EEA countries reporting consumption per quarter for at least 15 years, 1997-2017.**


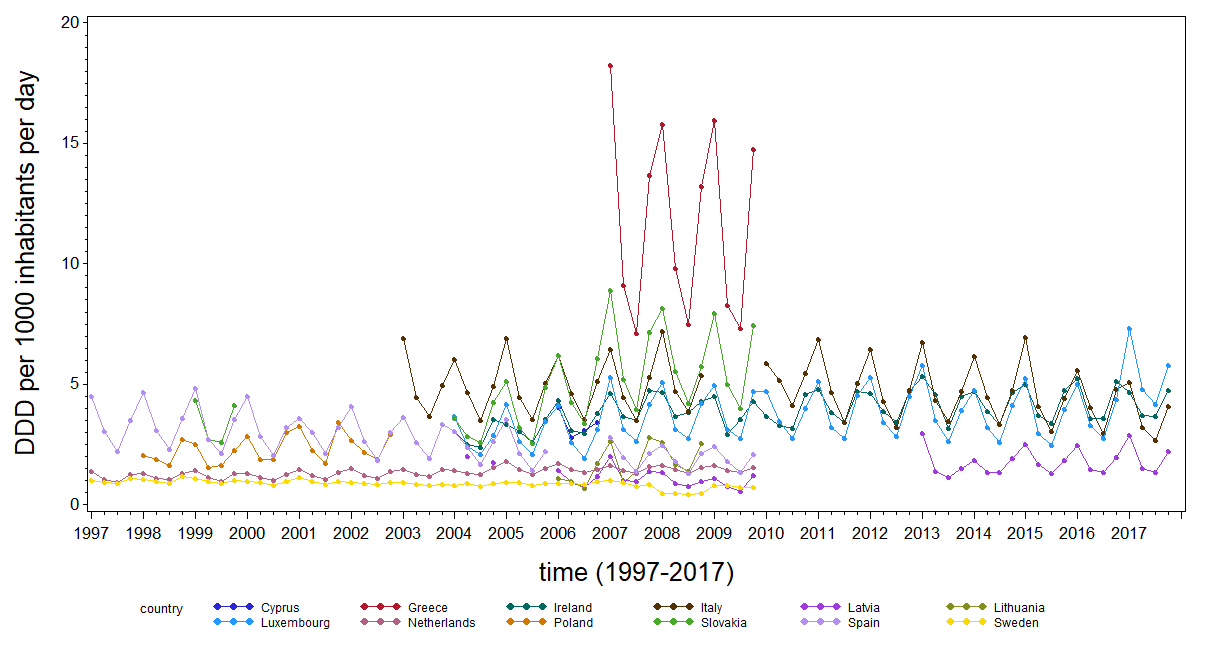


**Figure S3. Seasonal variation in consumption of macrolides, lincosamides and streptogramins (ATC J01F) in the community, expressed in DDD (ATC/DDD index 2019) per 1000 inhabitants per day, 12 EU countries reporting consumption per quarter for less than 15 years, 1997-2017. For Cyprus, total care data, i.e. community and hospital sector combined, are used. For Spain, private prescriptions are included from 2016 onwards.**

^
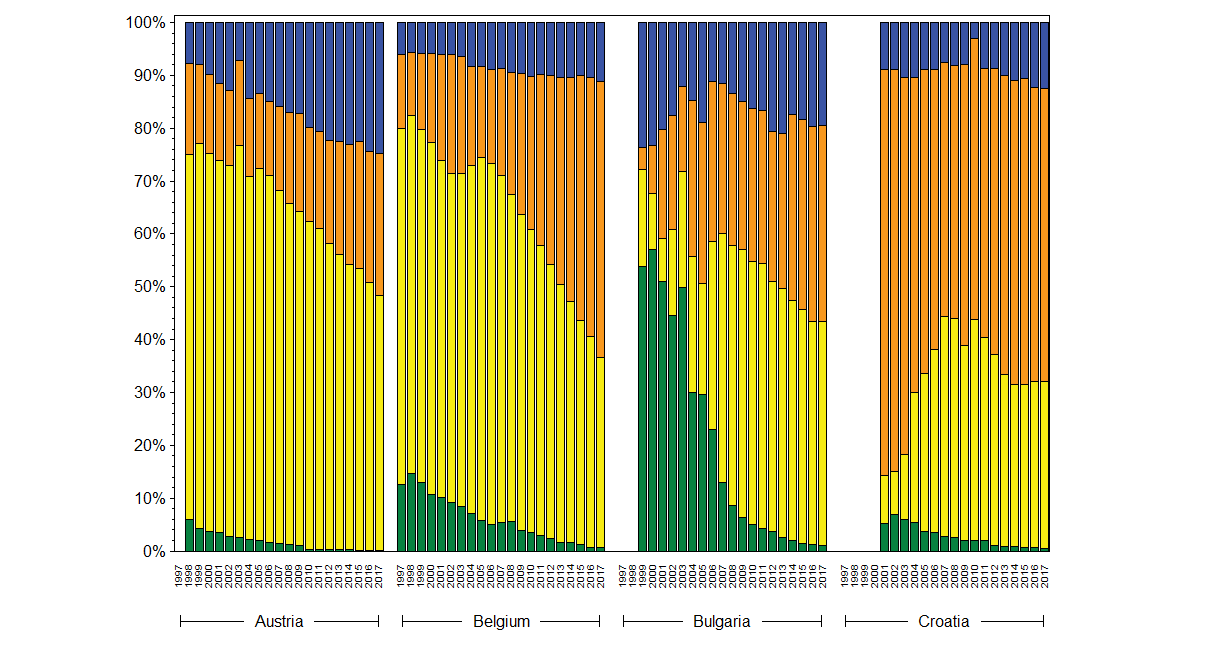
^

^
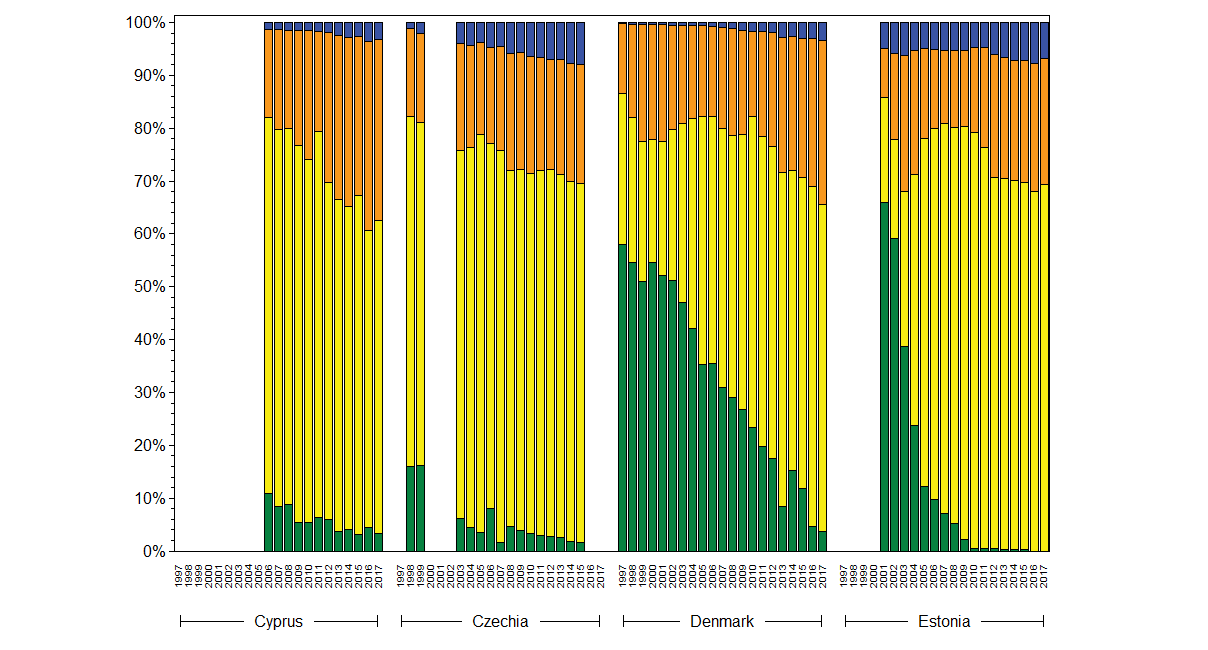
^

= short-acting macrolides, = intermediate-acting macrolides,

= long-acting macrolides, = lincosamides, = streptogramins

**Figure S4. Composition of macrolides, lincosamides and streptogramins (ATC J01F) consumption in the community, expressed in DDD (ATC/DDD index 2019) per 1000 inhabitants per day, 30 EU/EEA countries, 1997-2017. For Cyprus and Romania, total care data, i.e. community and hospital sector combined, are used. For Spain, private prescription are included from 2016 onwards. For Romania, data have a coverage in 2009 limited to 30-40%.**

^
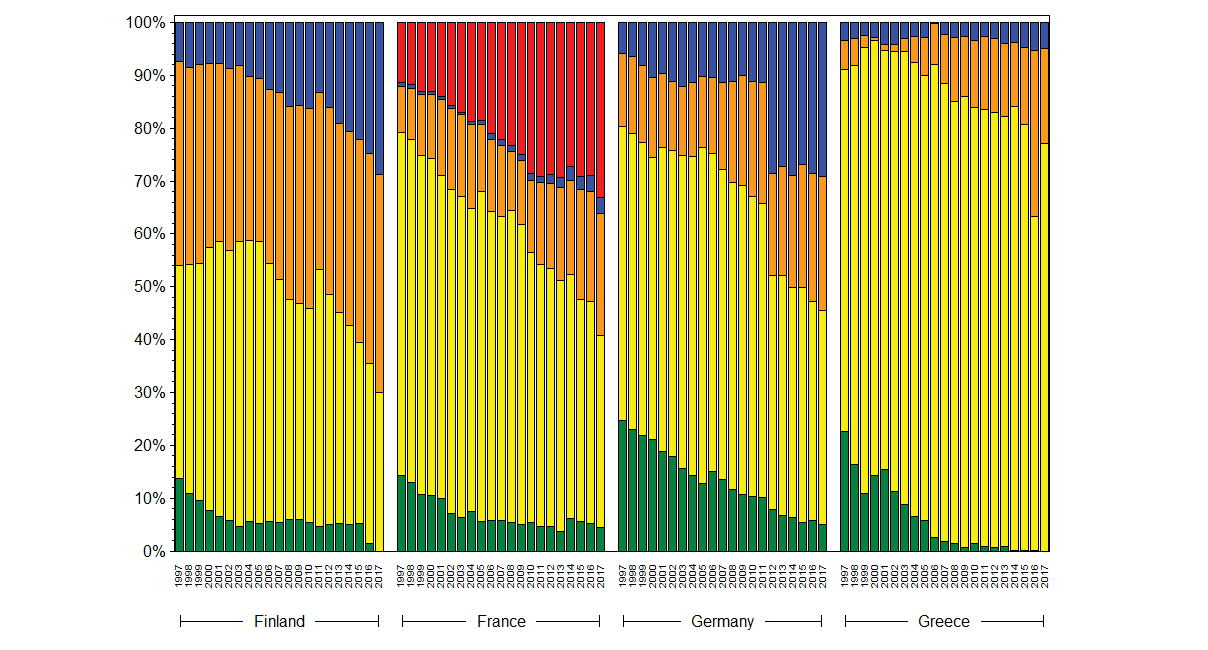

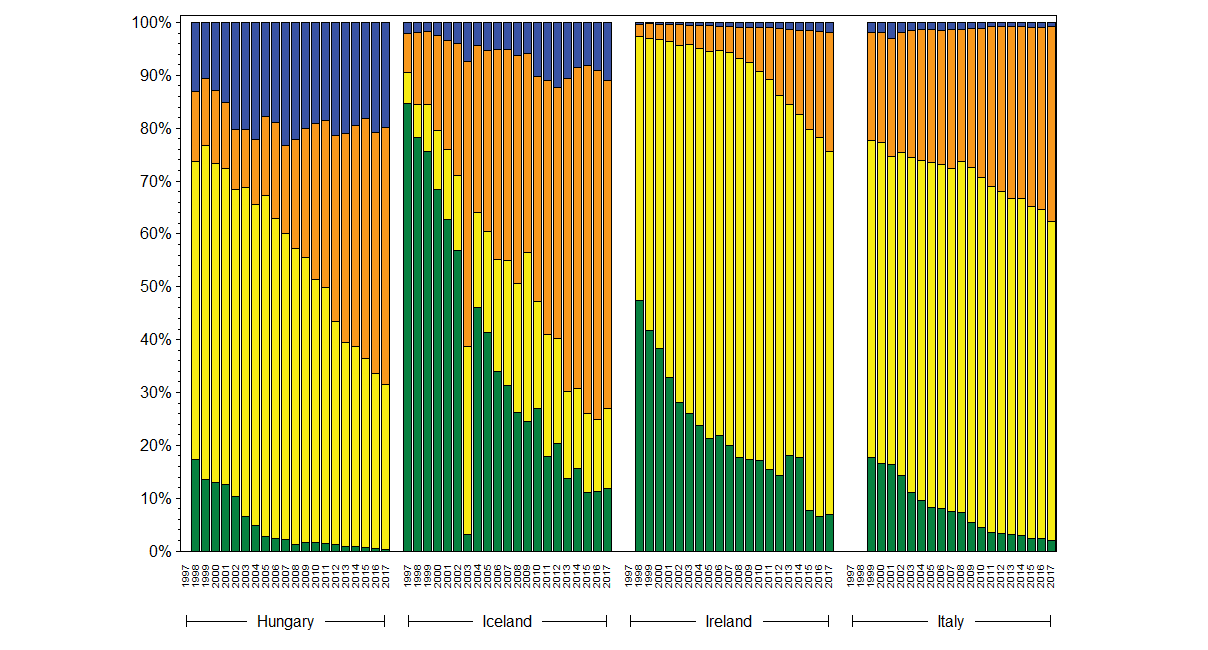
^

= short-acting macrolides, = intermediate-acting macrolides,

= long-acting macrolides, = lincosamides, = streptogramins

**Figure S4.** Continued

^
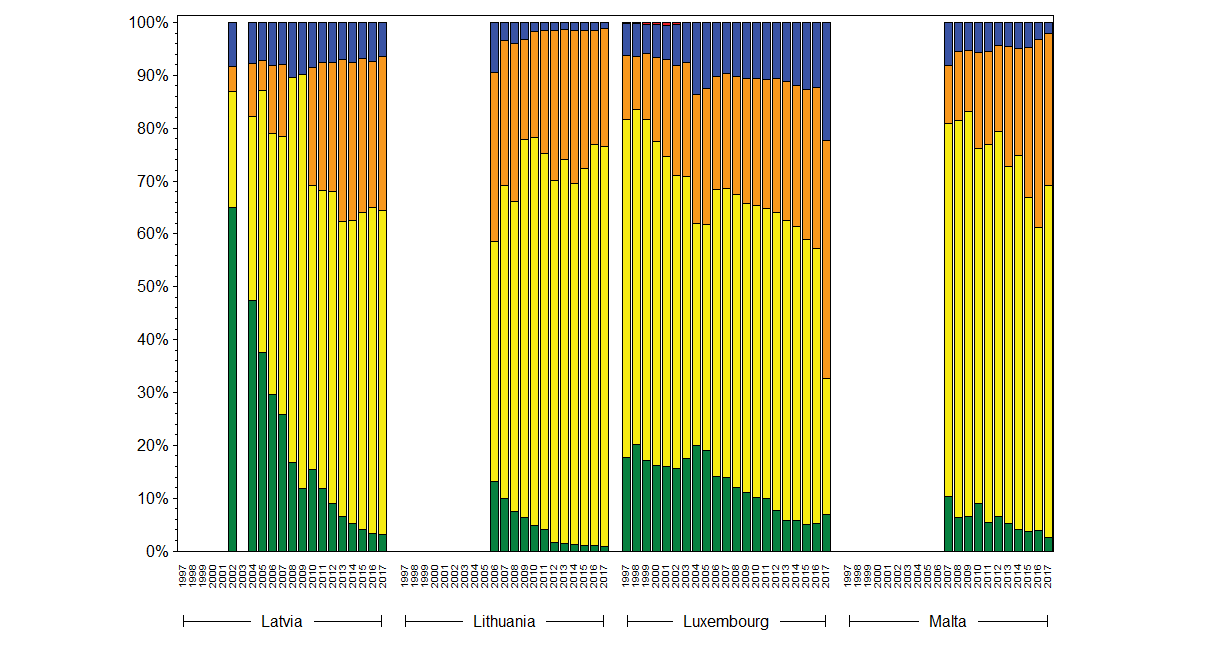
^

^
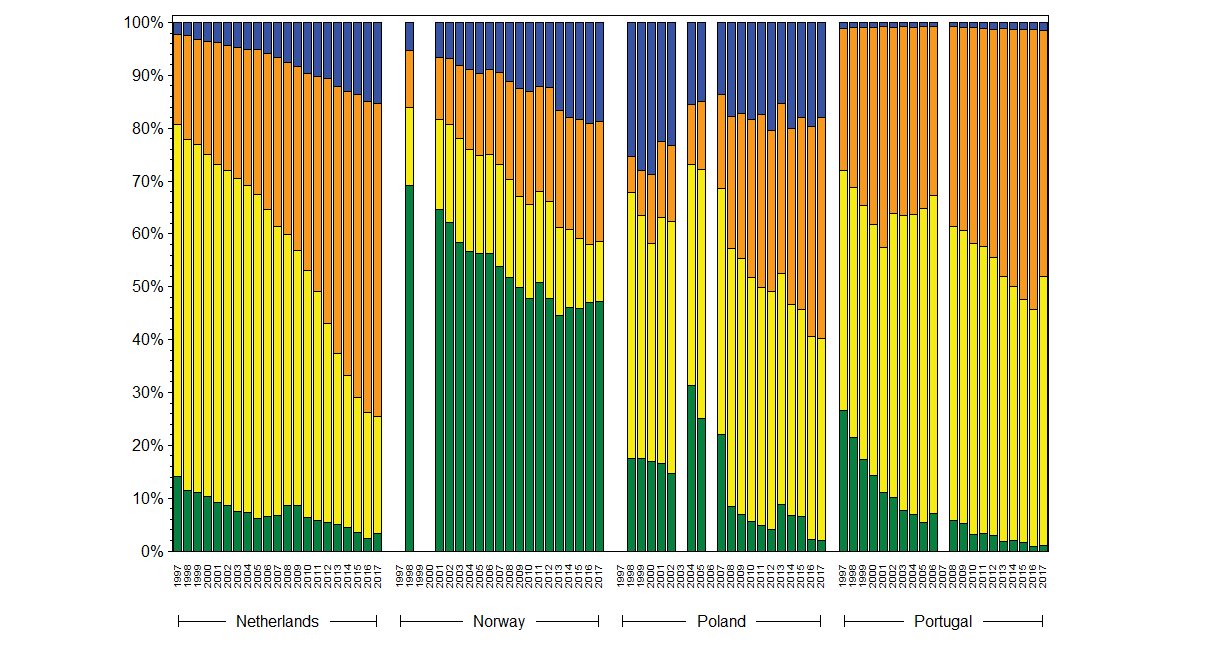
^

= short-acting macrolides, = intermediate-acting macrolides,

= long-acting macrolides, = lincosamides, = streptogramins

**Figure S4.** Continued

^
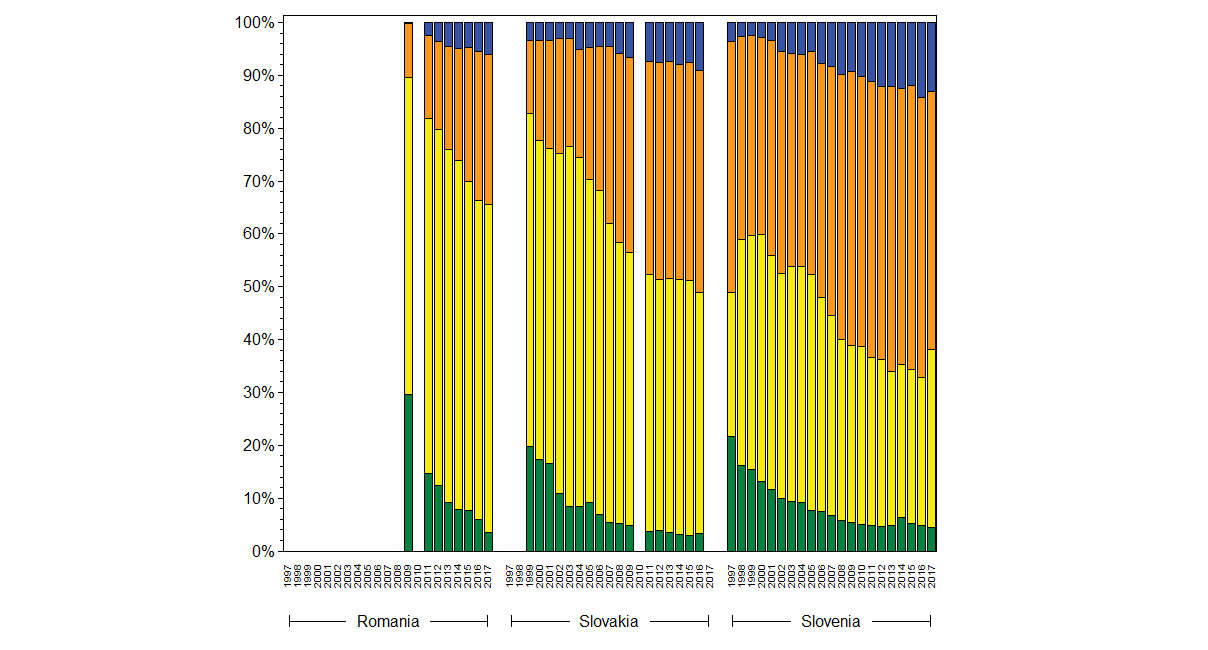
^


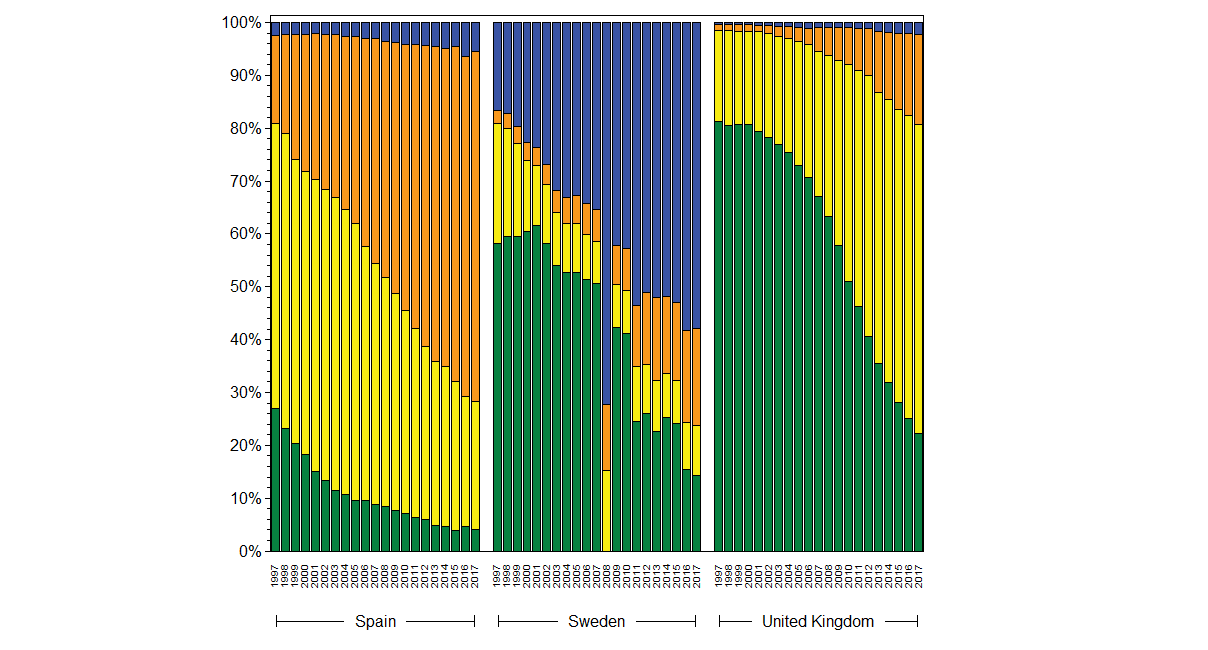


= short-acting macrolides, = intermediate-acting macrolides,

= long-acting macrolides, = lincosamides, = streptogramins

**Figure S4.** Continued
